# A Cross-sectional Survey of Physicians to Understand Biomarker Testing and Treatment Patterns in Patients with Prostate Cancer in the USA, EU5, Japan, and China

**DOI:** 10.1016/j.euros.2024.07.113

**Published:** 2024-10-10

**Authors:** Christian Gratzke, Himani Aggarwal, Jeri Kim, Holly Chaignaud, Sabine Oskar

**Affiliations:** a Department of Urology, University Hospital Freiburg, Freiburg, Germany; b Merck & Co. Inc, Rahway, NJ, USA; c OPEN Health, London, UK

**Keywords:** Genetic testing, Homologous recombination repair mutation, Metastatic castration-resistant prostate cancer, Poly-adenosine diphosphate-ribose polymerase inhibitors, Real-world data

## Abstract

**Background and objective:**

Treatment landscape in advanced prostate cancer (PC) is evolving. There is limited understanding of the factors influencing decision-making for genetic/genomic testing and the barriers to recommending testing and treatment in international real-world clinical practice following the approval of poly-adenosine diphosphate-ribose polymerase inhibitors (PARPi) for metastatic castration-resistant PC (mCRPC). This work aims to assess genetic/genomic testing patterns and methods, including for homologous recombination repair mutation (HRRm), and treatment decisions among physicians caring for patients with PC across the USA, Europe, and Asia.

**Methods:**

A cross-sectional online survey of physicians treating patients with advanced PC was administered in the USA, France, Germany, Italy, Spain, UK, Japan, and China. Physicians were recruited (from August to December 2022) via clinical panels and provided informed consent. Survey questions covered factors influencing HRRm testing and treatment decision-making.

**Key findings and limitations:**

Physicians reported that 50% of patients with mCRPC are recommended for HRRm testing, and among those recommended for testing, 60% are recommended for *BRCA1/2* mutation testing and 65% go on to receive HRRm testing. Overall proportions of patients recommended for testing increased following PARPi approval (from 20% to 50%) and following updated practice guidelines (from 25% to 50%). Perceived barriers to the use of genetic/genomic testing included patient refusal, lack of insurance/reimbursement, and lack of availability of adequate tissue for testing.

**Conclusions and clinical implications:**

Overall, testing rates increased following PARPi approval and updated clinical practice guidelines; yet, there was a wide variation in the proportions of patients with mCRPC recommended for testing, and perceived barriers to testing remain, suggesting unmet needs for patients and physicians.

**Patient summary:**

We surveyed physicians globally about their experience in treating patients with advanced prostate cancer and genetic testing. Physicians reported that half of patients are recommended for genetic testing, which varied across countries. We conclude that barriers to testing remain for patients and physicians.

## 1 Introduction

Globally, prostate cancer (PC) is the fifth leading cause of cancer deaths and the second most prevalent cancer in men [1]. Some patients with PC progress to metastatic hormone-sensitive PC (mHSPC) and others to nonmetastatic castration-resistant PC (nmCRPC). Of those who develop metastatic castration-resistant PC (mCRPC), about 30% will harbor deleterious alterations/mutations in genes including homologous recombination repair mutation (HRRm) [2], [3].

Until 2009, the standard of care for mCRPC included docetaxel and the combination of mitoxantrone and corticosteroids, and in 2010, sipuleucel-T and cabazitaxel were approved. Treatment for mCRPC has evolved in the last decade with the approval of novel therapies including next-generation hormonal agents (NHAs) and poly-adenosine diphosphate-ribose polymerase inhibitors (PARPi; olaparib and rucaparib) following pivotal trials [4], [5], [6]. NHAs (abiraterone, apalutamide, darolutamide, and enzalutamide) are approved in the USA, Europe, China, and Japan, and have been incorporated in clinical practice guidelines as standard of care in mCRPC, mHSPC, and nmCRPC [7], [8], [9], [10], [11], [12]. Rucaparib and olaparib are now approved in patients with mCRPC in the USA (May 2020) [13], [14], [15], and olaparib is approved in patients with mCRPC in Europe (November 2020) [16], the UK (May 2023) [17], Japan (December 2020) [18], and China (June 2021) [19].

Beyond informing prognosis and familial risk, PARPi approvals for mCRPC make genetic testing essential for treatment decisions [20], [21], [22]; clinical guidelines recommend genomic testing in this setting [7], [9], [10], [11], [12], [20], [23]. Previous data (from January to August 2020) reported that among observed patients with mCRPC, only 15.7% from selected European countries, 2.9% from Japan, and 38.2% from the USA had undergone HRRm testing before PARPi approval [22].

There is limited understanding of the factors underlying the decision-making process for genetic and genomic testing, as well as potential barriers to recommending testing and treatment in international real-world clinical practice following PARPi approval. To address this gap, we surveyed physicians treating patients with PC in the USA, Europe, Japan, and China about their use of biomarker testing, treatment patterns, and factors driving treatment decisions.

## 2 Materials and methods

### 2.1 Study design and data collection

This was a cross-sectional online survey among physicians treating patients with advanced PC (aPC) in the USA, EU5 (France, Germany, Italy, Spain, and the UK), Japan, and China. The survey was administered by Global Perspectives (Reading, UK) collaboratively with OPEN Health (London, UK). Participants were required to be an oncologist (inclusive of radiation and medical oncologists), a urologist, or a pathologist involved in the management and/or treatment of patients with mCRPC, nmCRPC, or mHSPC. Pathologists were required to be involved in conducting genomic/genetic testing for eligibility. Physician recruitment was proportional to the distribution of specialties involved in managing aPC in each country. All participants provided electronic consent, and the study was approved by the SALUS Institutional Review Board (January 24, 2022).

Physicians were recruited from online panels consisting of communities of preselected individuals interested in participating in online surveys. For consenting participants, a self-administered online screening form was completed to determine eligibility. Eligible physicians were subsequently sent an electronic link to the survey.

The survey was launched following a pilot phase consisting of 60-minute interviews in the local language with eight physicians (two each from the UK, the USA, France, and Japan) to ensure understanding of the survey’s purpose and the region-specific appropriateness of the questions. Feedback was used to revise the global survey, subsequently administered between August 30, 2022 and December 13, 2022. Survey topics included utilization of HRRm testing, considerations for adopting genetic/genomic testing, prescribing behavior (before/after PARPi approval and national clinical guideline updates), and factors influencing treatment decision-making for patients with nmCRPC, mHSPC, and mCRPC. The survey included core questions for all participants and some specialty-specific questions. Physicians were compensated (as per fair market value) upon completion of the survey.

### 2.2 Statistical methods

Physicians’ demographic and other characteristics were summarized, and responses for each survey question were analyzed descriptively. For categorical data, frequencies and percentages were calculated. For continuous data, measures of central tendency and dispersion were calculated, including mean, standard deviation, median, and interquartile ranges (IQRs). Responses to open-ended questions were reviewed, assigned to appropriate thematic categories, and summarized quantitatively. Skip logics were utilized to ask specific questions to respondents based on their answers to previous questions. The total number of responses per question is reported. Results are reported overall and stratified by country, specialty, and/or setting.

## 3 Results

### 3.1 Respondent characteristics

Of 1195 physician respondents, 34% (*n* = 407/1195) completed the survey: 100 were from the USA, 132 from EU5, 100 from Japan, and 75 from China. Overall, 40% (*n* = 164) were oncologists (38%, 1%, and 1% were medical, radiation, and surgical oncologists, respectively), 43% (*n* = 174) were urologists, and 17% (*n* = 69) were pathologists; the median (IQR) number of years in practice was 16.0 (11.5–22.0). About 59% (*n* = 239) practiced in academic centers, 30% (*n* = 123) in community centers, 8% (*n* = 31) in secondary care centers, and 3% (*n* = 14) in other settings. When asked about the proportion of their patients diagnosed with PC in the previous 3 years, 31% (*n* = 127/407) estimated that 21–40% had mCRPC, 37% (*n* = 150/407) estimated that ≤20% had nmCRPC, and 38% (*n* = 154/407) estimated that ≤20% had mHSPC (Supplementary Fig. 1).

### 3.2 Utilization pattern of HRRm testing in patients with aPC

The estimated proportions of patients with mCRPC who are recommended for and who receive HRRm testing are described in Table 1 by country and practice setting. Overall, physicians reported that 50% (IQR 20.0–75.0) of patients with mCRPC are recommended for HRRm testing, and among those recommended for HRRm testing, 60% (IQR 30.0–90.0) are recommended for *BRCA1/2* mutation testing and 65% (IQR 40.0–89.0) receive HRRm testing. Physicians practicing in academic centers reported recommending HRRm testing for a median of 50% (IQR 25.0–80.0) of mCRPC patients, while those in community centers reported 46% (IQR 20.0–78.8). Physicians in France and the UK reported recommending testing for 20% and 30% of patients, respectively, while physicians in Japan reported recommending 35% of patients for *BRCA1/2* mutation testing. Overall, physicians recommended testing at diagnosis of mCRPC (52%), at diagnosis of mHSPC (45%), or after first-line treatment for mCRPC (44%; Supplementary Fig. 2).

**Table 1:** HRRm testing patterns as reported by physicians overall and by region and practice setting

Overall			USA			EU5			China a		Japan		
All	Acad	Com	All	Acad	Com	All	Acad	Com	All	Acad	All	Acad	Com
(*n* = 407)	(*n* = 244)	(*n* = 122)	(*n* = 100)	(*n* = 33)	(*n* = 61)	(*n* = 132)	(*n* = 97)	(*n* = 20)	(*n* = 75)	(*n* = 59)	(*n* = 100)	(*n* = 55)	(*n* = 36)
*Proportion of patients with mCRPC who are recommended for HRRm testing, median (IQR)*													
50 (20.0–75.0)	50 (25.0–80.0)	45.5 (20.0–78.6)	67.5 (25.0–100.0)	70 (50.0–100.0)	50 (25.0–100.0)	35 (20.0–61.0)	40 (20.0–70.0)	30 (13.8–50.0)	60 (33.5–80.0)	60 (33.5–80.0)	40 (10.0–55.3)	40 (20.0–56.0)	25 (5.0–56.3)
*Of those recommended for HRRm testing, the proportion of patients with mCRPC who are recommended to be tested solely for BRCA1/2 mutations, median (IQR)*													
60 (30.0–90.0)	60 (30.0–90.0)	72.5 (25.0–100.0)	90 (50.0–100.0)	75 (60.0–100.0)	90 (50.0 – 100.0)	65 (30.0–90.0)	65 (30.0–90.0)	67.5 (28.8–100.0)	60 (30.0–80.0)	60 (32.5–80.0)	35 (15.0–66.3)	40 (25.0–60.0)	30 (3.8–80.0)
*Proportion of patients receiving HRRm testing (of mCRPC patients recommended to undergo HRRm testing), median (IQR)*													
65 (40.0–89.0)	65 (40.0–85.0)	75 (40.0–90.0)	80 (50.0–97.3)	90 (50.0–99.0)	80 (50.0–90.0)	72.5 (50.0–90.0)	80 (50.0–95.0)	72.5 (45.0–90.0)	60 (40.0–80.0)	65 (40.0–80.0)	42.5 (20.0–60.0)	45 (25.0–55.0)	50 (5.0–65.0)

Acad = academic; Com = community; EU5 = France, Germany, Italy, Spain, and UK; HRRm = homologous recombination repair mutation; IQR = interquartile range; mCRPC = metastatic castration-resistant prostate cancer; *n* = number of physicians that answered the survey question.

a Results are reported only for subgroups with more than five respondents. Results by country are available in Supplementary Table 1.

**Fig. 1:**
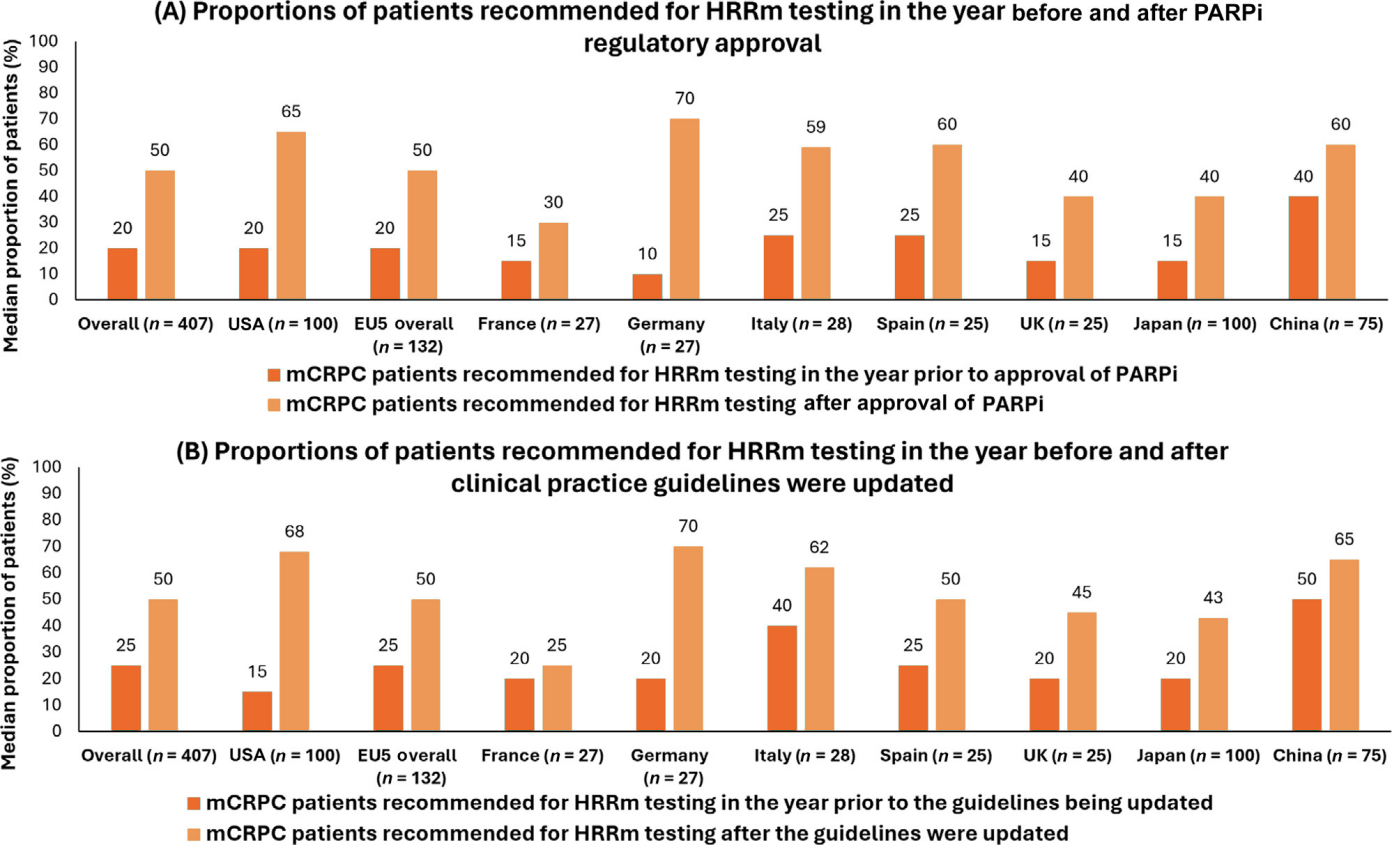
Proportions of patients with mCRPC recommended for HRRm testing in the year prior to and following (A) country-specific regulatory approval of PARPi and (B) the update of country-specific clinical practice guidelines. EU5 = France, Germany, Italy, Spain, and the UK; HRRm = homologous recombination repair mutation; mCRPC = metastatic castration-resistant prostate cancer; PARPi = poly-adenosine diphosphate-ribose polymerase inhibitor.

Overall, physicians reported that a median of 20% (IQR 5.0–45.0) of patients were recommended for HRRm testing in the year before regulatory approval of PARPi (PARPi US regulatory approval (US Food and Drug Administration) was achieved in May 2020 for olaparib and rucaparib, EU regulatory approval (European Medicines Agency) was achieved in November 2020 for olaparib, Japanese regulatory approval was achieved in December 2020 for olaparib, and Chinese regulatory approval was achieved in June 2021 for olaparib), while 50% (IQR 25.0–80.0) were recommended after approval (Fig. 1A). The smallest absolute increase in the proportion of patients with mCRPC being recommended for HRRm testing after approval was in France (15%/30% [before/after]), while the largest was in Germany (10%/70% [before/after]). Physicians in China reported the highest proportion of patients recommended for HRRm testing before approval (40%). Overall, physicians from academic settings reported an increase from 26% to 55% of patients, while those in community settings reported an increase from 10% to 50% (Supplementary Table 2).

**Fig. 2:**
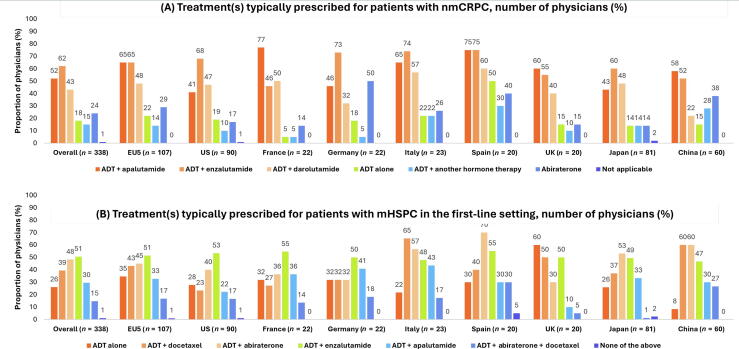
Treatments(s) typically prescribed for patients with nmCRPC and mHSPC overall and by country. ADT = androgen deprivation therapy; EU5 = France, Germany, Italy, Spain, and the UK; mHSPC = metastatic hormone-sensitive prostate cancer; nmCRPC = nonmetastatic castration-resistant prostate cancer.

Physicians reported that a median of 25% (IQR 7.0–50.0) of patients with mCRPC were recommended for HRRm testing the year before updated clinical guidelines (treatment guidelines were updated in 2019 in the USA by the National Comprehensive Cancer Network [NCCN], in 2021 in the EU by the European Association of Urology, in 2021 in Japan by the Japanese Urological Association, and in 2021 in China by the Chinese Society of Clinical Oncology) and 50% (IQR 25.0–80.0) after update (Fig. 2B). By country, the smallest absolute increase in the proportion of patients recommended for HRRm testing after updated guidelines was in France (20%/25% [before/after]), while the largest increases were in the USA (15%/68% [before/after]) and Germany (20%/70% [before/after]). Overall, physicians practicing in community settings reported a larger increase (20%/50% [before/after]) in patients being recommended after updated guidelines than in those in academic settings (30%/57% [before/after]; Supplementary Table 3).

Reported utilization of tissue biopsy/tumor testing, germline blood testing, and circulating tumor DNA (ctDNA) testing before and after PARPi approval is summarized in Supplementary Table 4. Overall, physicians reported that they “most frequently” recommended tissue biopsy/tumor testing both before (53%) and after PARPi (65%) approval. Overall, tissue biopsy/tumor testing and germline blood testing were indicated as being the first and second HRRm tests, respectively (according to 72% and 40% of physicians, respectively; Supplementary Fig. 3). Overall, the two most common reasons reported for choosing to start with tissue biopsy/tumor testing were higher accuracy and reliability (34%, *n* = 100/295), and availability and convenience of obtaining/accessing samples (19%, *n* = 55/295). Supplementary Table 5 summarizes the reasons driving the second testing type.

### 3.3 Factors affecting the adoption of HRRm testing

Table 2 summarizes the factors influencing HRRm testing recommendation and receipt, as reported by physicians. In the USA, EU5, China, and Japan, limited insurance coverage and patient refusal were the most common reasons for patients recommended for HRRm testing to not receive it. Overall, patient refusal was the most commonly reported challenge in adopting HRRm testing with tissue biopsy/tumor (41%) or germline blood samples (46%; Supplementary Fig. 4). Other common challenges for adopting tissue biopsy/tumor for HRRm testing, overall, included longer turnaround time to receive results (40%) and limited access to genetic counseling (40%).

**Table 2:** Patient characteristics and considerations affecting the recommendation and receipt of HRRm testing according to physicians overall and by country

	Overall	USA	EU5	France	Germany	Italy	Spain	UK	Japan	China
	(*n* = 407)	(*n* = 100)	(*n* = 132)	(*n* = 27)	(*n* = 27)	(*n* = 28)	(*n* = 25)	(*n* = 25)	(*n* = 100)	(*n* = 75)
*Patient characteristics that drive the clinical decision to recommend HRRm testing*^a^										
Young age at diagnosis of PC, *n* (%)	233 (57)	53 (53)	80 (61)	14 (52)	13 (48)	20 (71)	16 (64)	17 (68)	50 (50)	50 (67)
Family history of PC, *n* (%)	297 (73)	62 (62)	95 (72)	19 (70)	16 (59)	19 (68)	22 (88)	19 (76)	75 (75)	65 (87)
Family history of breast and ovarian cancer, *n* (%)	209 (51)	43 (43)	75 (57)	15 (56)	11 (41)	16 (57)	17 (68)	16 (64)	50 (50)	41 (55)
Patient with metastatic disease, *n* (%)	210 (52)	54 (54)	68 (52)	10 (37)	14 (52)	18 (64)	14 (56)	12 (48)	39 (39)	49 (65)
Patient has insurance coverage, *n* (%)	106 (26)	23 (23)	26 (20)	2 (7)	6 (22)	7 (25)	8 (32)	3 (12)	33 (33)	24 (32)
Progressing disease, *n* (%)	190 (47)	40 (40)	54 (41)	9 (33)	13 (48)	15 (54)	8 (32)	9 (36)	43 (43)	53 (71)
Failure of first-line therapy, *n* (%)	153 (38)	44 (44)	47 (36)	8 (30)	11 (41)	13 (46)	8 (32)	7 (28)	19 (19)	43 (57)
Other, *n* (%)	7 (2)	5 (5)	1 (1)	0 (0)	0 (0)	1 (4)	0 (0)	0 (0)	1 (1)	0 (0)
Not applicable b,*n* (%)	34 (8)	19 (19)	7 (5)	2 (7)	3 (11)	0 (0)	1 (4)	1 (4)	6 (6)	2 (3)
*Patient characteristics related to why patients who are recommended for HRRm testing do not undergo the testing*^a^										
Patient concerns of the privacy of genetic testing results/data selling and sharing policies of genetic labs, *n* (%)	142 (35)	22 (22)	46 (35)	6 (22)	5 (19)	13 (46)	9 (36)	13 (52)	41 (41)	33 (44)
Limited insurance coverage of the patient and out-of-pocket costs for the test, *n* (%)	224 (55)	63 (63)	51 (39)	7 (26)	11 (41)	14 (50)	9 (36)	10 (40)	44 (44)	66 (88)
Patient concerns about genetic discrimination, *n* (%)	118 (29)	23 (23)	39 (30)	8 (30)	5 (19)	7 (25)	9 (36)	10 (40)	35 (35)	21 (28)
Patient refusal, *n* (%)	203 (50)	50 (50)	57 (43)	11 (41)	12 (44)	15 (54)	5 (20)	14 (56)	50 (50)	46 (61)
Other, *n* (%)	12 (3)	4 (4)	5 (4)	0 (0)	1 (4)	2 (7)	2 (8)	0 (0)	1 (1)	2 (3)
Not applicable b,*n* (%)	49 (12)	19 (19)	20 (15)	6 (22)	6 (22)	1 (4)	4 (16)	3 (12)	10 (10)	0 (0)

EU5 = France, Germany, Italy, Spain, and UK; HRRm = homologous recombination repair genes; PC = prostate cancer.

a Responses were not mutually exclusive (respondents selected all options that applied). The responses provided are estimates based on physician reporting.

b Physicians reported that either HRRm testing was undertaken for all patients or they were unable to recommend testing due to a lack of reimbursement.

### 3.4 Treatment patterns among patients with mCRPC

Overall, decision to treat with PARPi was primarily influenced by HRRm status (41%) and treatment efficacy (40%), with variations observed across countries (Supplementary Table 6). Physicians in both China (40%) and Japan (25%) reported fewer and less severe comorbidities more commonly than HRRm status (37% and 22%, respectively) as a factor influencing decision to treat with PARPi. Overall, physicians reported that a median of 50% and 45% of patients with mCRPC received NHAs at first and second line, respectively (Table 3). Factors influencing physician’s decision to treat with NHAs or chemotherapy are summarized in Supplementary Table 6.

**Table 3:** Proportion of patients with mCRPC receiving NHAs or taxane-based chemotherapy in the first- and second-line setting overall and by country

	Overall a (*n* = 338)	USA (*n* = 90)	EU5 (*n* = 107)	France (*n* = 22)	Germany (*n* = 22)	Italy (*n* = 23)	Spain (*n* = 20)	UK (*n* = 20)	Japan (*n* = 81)	China (*n* = 60)
*Proportion receiving NHAs among patients with mCRPC in the first-line setting*										
Median (IQR)	50 (25–80)	75 (46–90)	65 (30–80)	73 (53–91)	63 (46–80)	50 (35–80)	70 (19–80)	55 (29–71)	35 (10–60)	40 (20–60)
*Proportion receiving NHAs among patients with mCRPC in the second-line setting*										
Median (IQR)	45 (25–50)	50 (25–70)	50 (30–60)	50 (20–69)	50 (31–64)	50 (30–62)	30 (20–53)	40 (20–50)	40 (20–70)	40 (29–60)
*Proportion receiving taxane-based chemotherapy among patients with mCRPCin the first-line setting*										
Median (IQR)	29 (10–50)	20 (10–35)	30.0 (20–50)	20.0 (16–45)	30 (20–48)	30 (20–55)	20 (13–40)	30 (20–39)	20 (5–40)	45 (30–55)
*Proportion receiving taxane-based chemotherapy among patients with mCRPC in the second-line setting*										
Median (IQR)	35 (20–50)	40 (25–60)	40.0 (25–60)	50.0 (23–60)	40 (30–50)	40 (28–61)	43 (29–61)	35 (20–51)	30 (15–50)	40 (30–60)

EU5 = France, Germany, Italy, Spain, and UK; mCRPC = metastatic castration-resistant prostate cancer; NHA = next-generation hormonal agent.

a Results include only urologists and oncologists.

### 3.5 Treatment patterns among patients with nmCRPC and mHSPC

Overall, physicians reported that a median (IQR) of 50% (30.0–80.0%) of patients with nmCRPC receive NHAs and 30% (15.0–50.0%) receive androgen deprivation therapy (ADT) + apalutamide. For patients with mHSPC, physicians reported that a median (IQR) of 50% (25.0–70.0%) receive NHAs and 30% (15.0–50.0%) receive ADT + enzalutamide. Findings were broadly consistent across countries. Treatments that physicians reported typically prescribing in the first-line setting for nmCRPC and mHSPC are summarized overall and by country in Figures 2A and 2B, respectively. Characteristics associated with the physician’s decision to treat patients with NHAs are summarized in Supplementary Tables 7 and 8.

## 4 Discussion

This global survey aimed to collect information on biomarker testing (including HRRm), treatment patterns, and factors affecting treatment decisions among physicians caring for patients with aPC across the USA, Europe, and Asia. Overall, physicians estimated a 30 percentage point increase in the proportion of patients recommended for HRRm testing in the year following regulatory approval of PARPi compared with the prior year. Physicians reported a wide variation in the percentage of patients with mCRPC recommended for HRRm testing and the timing in the disease course when they recommended testing. This study expands on previous evidence by providing information on HRRm testing trends after PARPi approval and clinical guideline updates, in addition to the reasons driving prescribing behavior and the inclusion of academic centers.

Overall, physicians reported that 50% of patients with mCRPC were estimated to have been recommended for HRRm testing, higher than the 18.1% reported in a previous survey (averaged across the USA, EU5, and Japan) and higher than a USA survey that reported testing in only 38% of patients with mCRPC (data from both studies were recorded between January and August 2020) [22], [24]. A study of electronic medical records in the USA observed that while HRRm testing in mCRPC increased between 2013 and 2018, only 13% of patients received genetic testing [25]. In our study, physicians reported a slightly higher proportion (20%) of patients recommended for HRRm testing in the year prior to the USA PARPi approval (May 2020) possibly reflecting the 2019 NCCN guideline updates that recommend the use of germline and somatic testing panels including HRR genes [7], [23]. Physicians in China reported the highest proportion of patients recommended for testing before PARPi approval, likely due to 2018 national guidelines that recommended germline tests for patients with metastatic PC [11]. In EU5, physicians in France and the UK reported lower proportions of patients recommended for HRRm testing (20% and 30%, respectively) than those in Germany (65%), despite having the same clinical practice guidelines [9]. France showed the smallest increase in the proportion of patients recommended for HRRm testing following the updated guidelines, while Germany observed the largest increase. This difference is likely due to variations in reimbursement dates, with France receiving reimbursement in November 2022 and Germany in November 2020. Physicians in Japan reported a low proportion (35%) of patients tested for *BRCA* mutations, possibly due to a greater prevalence of *ATM* testing and limited physician awareness of testing and educational resources, which were cited as challenges related to HRRm testing rates in a prior study [22].

The observed proportions recommended for testing may relate to the reimbursement status of PARPi in their respective healthcare systems and subsequently; hence, testing may increase once reimbursement is achieved. New PARPi approvals in the first-line settings for patients with mCRPC have occurred since this survey was completed in 2022 [15], [26], [27]; future studies may assess the impact of these approvals on HRRm testing and treatment patterns for patients with aPC, and the influence of insufficient HRRm testing on treatment patterns.

Perceived barriers to utilizing genetic and genomic testing included patient refusal, lack of insurance/reimbursement, and lack of availability of adequate tissue for testing, which align with the findings of previous studies and highlight ongoing barriers that remain to be addressed [28]. Results suggest that there may be an unmet need for patients and limited physician resources related to longer turnaround time to receive results and limited access to genetic counseling to facilitate the adoption of HRRm testing.

Treatment efficacy and the presence of fewer comorbidities were reported as the most common factors influencing the decision to treat mCRPC, nmCRPC, and mHSPC with NHAs. The majority of patients with nmCRPC were reported to be receiving NHAs, most commonly ADT + apalutamide or ADT + enzalutamide in line with current guidance [29]. For patients with mHSPC, the most frequently reported first-line treatments were ADT + enzalutamide, ADT + abiraterone, and ADT + docetaxel. These results differ somewhat from those published; the overall estimated proportion of patients with mHSPC receiving NHAs in the present study is higher at 50% than 30.7% initiated on NHA (±ADT), and the reported proportions receiving abiraterone, enzalutamide, and apalutamide were all higher in this study (30%, 30%, and 20%, respectively) than reported previously (20.7%, 8.2%, and 1.7%, respectively) [30].

This study had limitations. The results are based on a nonrandom convenience sample of physicians, which may introduce a selection bias as participating physicians may not represent those who opt out or do not respond. Recall bias, a common survey limitation, may affect the accuracy of responses as physicians have to provide approximate proportions from memory. Although the sample size for some countries was low, the responses from different countries were relatively homogeneous. Oncologists, urologists, and pathologists were recruited to be representative of the relevant proportions involved in treating patients with aPC; the low number of pathologists means that no inferences can be made specifically around that specialty. A major strength of this study is that it provides important insight into the real-world practice and experiences of physicians across the globe involved in the treatment of aPC, across specialties and practice settings.

## 5 Conclusions

Recommendations for HRRm testing increased following PARPi regulatory approval and updated clinical guidelines; yet, there was a wide variation in the proportions of patients with mCRPC recommended for testing and in the disease stage at which the test was recommended, as reported by physicians. Common barriers to HRRm testing included patient refusal, lack of reimbursement, and lack of availability of adequate tissue for testing. The reported lack of availability of adequate tissue for testing may present an opportunity for increased ctDNA testing.

  ***Author contributions*:** Christian Gratzke had full access to all the data in the study and takes responsibility for the integrity of the data and the accuracy of the data analysis.

  *Study concept and design:* Oskar, Aggarwal, Kim.

*Acquisition of data:* Oskar, Aggarwal, Kim.

*Analysis and interpretation of data:* Chaignaud, Oskar, Aggarwal, Kim, Gratzke.

*Drafting of the manuscript:* Oskar, Aggarwal, Kim, Gratzke.

*Critical revision of the manuscript for important intellectual content:* Chaignaud, Oskar, Aggarwal, Kim, Gratzke.

*Statistical analysis:* Chaignaud.

*Obtaining funding:* Oskar, Aggarwal, Kim.

*Administrative, technical, or material support:* Oskar, Aggarwal, Kim.

*Supervision:* Oskar, Aggarwal, Kim.

*Other:* None.

  ***Financial disclosures:*** Christian Gratzke certifies that all conflicts of interest, including specific financial interests and relationships and affiliations relevant to the subject matter or materials discussed in the manuscript (eg, employment/affiliation, grants or funding, consultancies, honoraria, stock ownership or options, expert testimony, royalties, or patents filed, received, or pending), are the following: Professor Dr. Christian Gratzke reports receipt of grants and/or contracts from AstraZeneca, Bayer, and Merck Sharp & Dohme LLC, a subsidiary of Merck & Co., Inc., Rahway, NJ, USA; receipt of consulting fees from Astellas Pharma, AstraZeneca, Bayer, Janssen, STEBA Biotech, and MSD; receipt of payment or honoraria from Amgen, Astellas Pharma, AstraZeneca, Bayer, GlaxoSmithKline, Ipsen, Janssen, Lilly Pharma, MSD, Pfizer, and STEBA Biotech; receipt of payment for expert testimony from Amgen, Astellas Pharma, AstraZeneca, Bayer, GlaxoSmithKline, Ipsen, Janssen, Lilly Pharma, MSD, and Pfizer; receipt of support for meetings/travel from Astellas Pharma, AstraZeneca, Bayer, Ipsen, and Janssen; participation in safety/advisory boards for Amgen, Astellas Pharma, AstraZeneca, Bayer, GlaxoSmithKline, Ipsen, Janssen, Lilly Pharma, MSD, Pfizer, and STEBA Biotech; and receipt of equipment, materials, drugs, medical writing, gifts, or other services from Merck Sharp & Dohme LLC, a subsidiary of Merck & Co., Inc., Rahway, NJ, USA. Dr. Sabine Oskar reports employment with Merck Sharp & Dohme LLC, a subsidiary of Merck & Co., Inc., Rahway, NJ, USA, and stock ownership of Merck & Co., Inc., Rahway, NJ, USA. Dr. Jeri Kim was formerly employed by Merck Sharp & Dohme LLC, a subsidiary of Merck & Co., Inc., Rahway, NJ, USA, and held stock of Merck & Co., Inc., Rahway, NJ, USA. At the time this study was conducted, Holly Chaignaud was employed by OPEN Health as a Data Analyst who were contracted by Merck & Co., Inc. for activities related to data collection and analysis in this study; she ceased working for the company in July 2023. Dr. Himani Aggarwal reports employment with Merck Sharp & Dohme LLC, a subsidiary of Merck & Co., Inc., Rahway, NJ, USA; stock ownership of Merck & Co., Inc., Rahway, NJ, USA; and stock ownership of Eli Lilly and Co.

  ***Funding/Support and role of the sponsor*:** This study was supported by Merck Sharp & Dohme LLC, a subsidiary of Merck & Co., Inc., Rahway, NJ, USA, and AstraZeneca UK Limited, which are codeveloping olaparib. The sponsor played a role in the design and conduct of the study; collection and management, analysis, and interpretation of the data; and preparation, review and approval of the manuscript.

  ***Acknowledgments*:** The authors would like to thank Alicia Gayle, PhD, for her support in the conduct of the study. Data analysis support was provided by Chloe Middleton-Dalby of OPEN Health, London, and medical writing support was provided by Will Cottam of OPEN Health, London.
