# Supplementary material for: A Cross-sectional Survey of Physicians to Understand Biomarker Testing and Treatment Patterns in Patients with Prostate Cancer in the USA, EU5, Japan, and China
Source: Eur Urol Open Sci. 2024 Oct 10;71:148–55. doi: 10.1016/j.euros.2024.07.113 (PMC11751505; doi:10.1016/j.euros.2024.07.113)
Supplement: Supplementary Data 1 [file mmc1.docx]

**Supplementary material**

**A Cross-Sectional Survey of Physicians to Understand Biomarker Testing and Treatment Patterns in Patients with Prostate Cancer in the USA, EU5, Japan, and China**

Christian Gratzke, Himani Aggarwal, Jeri Kim, Holly Chaignaud, Sabine Oskar

**Supplementary Table 1. HRRm testing patterns as reported by physicians overall and by country and practice setting in Europe**

| **Region** | **France** | | | | **Germany** | | **Italy** | | **Spain** | | | **UK** | | |
| --- | --- | --- | --- | --- | --- | --- | --- | --- | --- | --- | --- | --- | --- | --- |
| **Practice setting*** | **All** | **Acad** | **Com** | **All** | | **Acad** | **All** | **Acad** | | **All** | **Acad** | | **All** | **Acad** |
|  | **n=27** | **n=17** | **n=7** | **n=27** | | **n=18** | **n=28** | **n=20** | | **n=25** | **n=21** | | **n=25** | **n=21** |
| **Proportion of patients with mCRPC who are recommended for HRRm testing, median (IQR)** | | | | | | | | | | | | | | |
|  | 67.5 (25.0-100.0) | 70 (50.0-100.0) | 50 (25.0-100.0) | 65 (27.5- 85.0) | | 80 (35.0- 90.0) | 47.5 (28.8- 66.8) | 52.5 (34.8- 75.0) | | 40 (20.0- 50.0) | 40 (20.0- 50.0) | | 30 (10.0- 50.0) | 30 (10.0- 50.0) |
| **Of those recommended for HRRm testing, the proportion of patients with mCRPC who are recommended to be tested for *BRCA1/2* mutations testing, median (IQR)** | | | | | | | | | | | | | | |
|  | 90 (50.0- 100.0) | 75 (60.0- 100.0) | 90 (50.0 – 100.0) | 90 (50.0- 97.5) | | 90 (57.5- 95.0) | 65 (48.8- 80.0) | 63.5 (48.8- 80.0) | | 70 (35.0- 90.0) | 80 (35.0- 90.0) | | 40 (30.0- 90.0) | 40 (30.0- 90.0) |
| **Proportion of patients receiving HRRm testing (of mCRPC patients recommended to undergo HRRm testing), median (IQR)** | | | | | | | | | | | | | | |
|  | 80 (50.0- 97.3) | 90 (50.0- 99.0) | 80 (50.0- 90.0) | 80 (55.0- 90.0) | | 82.5 (70.0- 90.0) | 80 (50.0- 90.0) | 80 (50.0- 90.0) | | 75 (50.0- 95.0) | 75 (50.0- 95.0) | | 60 (50.0- 85.0) | 70 (50.0- 90.0) |

*N represents the number of physicians that answered the survey question. **Results reported only for subgroups with >5 respondents. Key: Acad – academic, Com – community, HRRm – homologous recombination repair mutation, IQR – interquartile range, mCRPC – metastatic castration-resistant prostate cancer, UK – United Kingdom.

**Supplementary Table 2. Proportions of patients with mCRPC recommended for HRRm testing in the year prior to and following regulatory approval of PARPi, by country and setting**

| **Overall** | | | | | | | | | | |
| --- | --- | --- | --- | --- | --- | --- | --- | --- | --- | --- |
| **By Country** | **Overall (n=407)** | **EU5 Overall (n=132)** | **US**  **(n=100)** | **France (n=27)** | **Germany (n=27)** | **Italy**  **(n=28)** | **Spain (n=25)** | **UK**  **(n=25)** | **Japan (n=100)** | **China (n=75)** |
| **Proportion of patients tested in year prior to regulatory approval of PARP inhibitors** | 20 (5.0- 45.0) | 20 (3.0- 40.0) | 20 (5.0- 40.0) | 15 (0.0- 20.0) | 10 (3.50- 40.0) | 25 (5.0- 50.0) | 25 (10.0- 60.0) | 15 (2.0- 30.0) | 15 (0.0- 40.0) | 40 (20.0- 60.0) |
| **Proportion of patients tested in year after regulatory approval of PARP inhibitors** | 50 (25.0- 80.0) | 50 (25.0- 76.3) | 64.5 (25.0- 91.3) | 30 (15.0- 55.0) | 70 (35.0 - 80.0) | 59 (38.8- 70.0) | 60 (25.0 - 90.0) | 40 (20.0- 50.0) | 40 (17.5- 60.0) | 60 (42.5- 80.0) |
| **Academic setting** | | | | | | | | | | |
|  | **Overall (n=244)** | **EU5 Overall (n=97)** | **USA**  **(n=33)** | **France (n=17)** | **Germany (n=18)** | **Italy**  **(n=20)** | **Spain (n=21)** | **UK**  **(n=21)** | **Japan (n=55)** | **China (n=59)** |
| **Proportion of patients tested in year prior to regulatory approval of PARP inhibitors** | 26 (9.5 - 50.0) | 20 (5.0- 43.0) | 25 (5.0- 50.0) | 15 (0.0- 20.0) | 17.5 (5.0- 47.5) | 25 (8.8- 53.8) | 25 (5.0- 60.0) | 10 (2.0- 30.0) | 30 (10.0- 40.0) | 40 (20.0- 60.0) |
| **Proportion of patients tested in time after regulatory approval of PARP inhibitors** | 55 (30.0- 80.0) | 50 (25.0- 80.0) | 80 (50.0- 95.0) | 40 (20.0- 70.0) | 80 (32.5- 83.8) | 59 (47.5- 70.0) | 60 (25.0- 90.0) | 30 (20.0- 50.0) | 40 (24.0- 60.0) | 60 (45.0- 80.0) |
| **Community setting** | | | | | | | | | | |
|  | **Overall (n=122)** | **EU5 Overall (n=20)** | **USA**  **(n=61)** | **France (n=7)** | **Germany*** | **Italy*** | **Spain*** | **UK*** | **Japan (n=36)** | **China*** |
| **Proportion of patients tested in year prior to regulatory approval of PARP inhibitors** | 10 (0.0– 29.0) | 20 (0.0– 36.3) | 15 (5.0– 25.0) | 0 (0.0– 17.5) |  |  |  |  | 0 (0.0– 20.0) |  |
| **Proportion of patients tested in time after regulatory approval of PARP inhibitors** | 50 (20.0– 80.0) | 50 (25.0– 63.8) | 50 (25.0– 90.0) | 25 (8.0 – 40.0) |  |  |  |  | 40 (8.8– 60.0) |  |
| **Secondary setting** | | | | | | | | | | |
|  | **Overall (n=22)** | **EU5 Overall (n=13)** | **USA*** | **France*** | **Germany*** | **Italy*** | **Spain*** | **UK*** | **Japan*** | **China*** |
| **Proportion of patients tested in year prior to regulatory approval of PARP inhibitors** | 18 (5.0- 41.3) | 15 (5.0- 20.0) |  |  |  |  |  |  |  |  |
| **Proportion of patients tested in time after regulatory approval of PARP inhibitors** | 35 (16.3- 60.0) | 25 (20.0- 50.0) |  |  |  |  |  |  |  |  |

*Results reported only for subgroups with >5 respondents. Key: EU5 – overall results from across France/Germany/Italy/Spain/UK, HRRm – homologous recombination repair mutation, IQR – interquartile range, mCRPC – metastatic castration-resistant prostate cancer, PARPi – poly-adenosine diphosphate-ribose polymerase inhibitor, United Kingdom, US – United States.

**Supplementary Table 3. Proportions of patients with mCRPC recommended for HRRm testing in the year prior to and following updated clinical practice guidelines, by country and setting**

| **Overall** | | | | | | | | | | |
| --- | --- | --- | --- | --- | --- | --- | --- | --- | --- | --- |
| **By Country** | **Overall (n=407)** | **EU5 Overall (n=132)** | **USA**  **(n=100)** | **France (n=27)** | **Germany (n=27)** | **Italy**  **(n=28)** | **Spain (n=25)** | **UK**  **(n=25)** | **Japan (n=100)** | **China (n=75)** |
| **Proportion of patients tested in year prior to updated guidelines** | 25.00 (7.00 - 50.00) | 25.00 (10.00 - 56.25) | 15.00 (0.00 - 40.00) | 20.00 (7.50 - 32.50) | 20.00 (10.00 - 50.00) | 40.00 (20.00 - 70.00) | 25.00 (5.00 - 75.00) | 20.00 (5.00 - 30.00) | 20.00 (0.00 - 42.75) | 50.00 (22.50 - 62.50) |
| **Proportion of patients tested in year after updated guidelines** | 50.00 (25.00 - 80.00) | 50.00 (20.00 - 80.00) | 67.50 (25.00 - 90.00) | 25.00 (20.00 - 75.00) | 70.00 (42.50 - 85.00) | 61.50 (47.50 - 75.00) | 50.00 (25.00 - 75.00) | 45.00 (20.00 - 50.00) | 42.50 (18.75 - 60.00) | 65.00 (40.00 - 80.00) |
| **Academic setting** | | | | | | | | | | |
|  | **Overall (n=244)** | **EU5 Overall (n=97)** | **USA**  **(n=33)** | **France (n=17)** | **Germany (n=18)** | **Italy**  **(n=20)** | **Spain (n=21)** | **UK**  **(n=21)** | **Japan (n=55)** | **China (n=59)** |
| **Proportion of patients tested in year prior to updated guidelines** | 30.00 (10.00 - 60.00) | 25.00 (10.00 - 70.00) | 20.00 (0.00 - 50.00) | 25.00 (10.00 - 60.00) | 32.50 (12.50 - 66.25) | 42.50 (20.00 - 71.25) | 35.00 (5.00 - 75.00) | 20.00 (5.00 - 30.00) | 30.00 (15.00 - 47.50) | 50.00 (25.00 - 63.00) |
| **Proportion of patients tested in year after updated guidelines** | 57.00 (30.00 - 80.00) | 50.00 (25.00 - 80.00) | 70.00 (50.00 - 95.00) | 40.00 (20.00 - 80.00) | 80.00 (41.25 - 88.75) | 57.00 (47.50 - 71.25) | 50.00 (20.00 - 75.00) | 30.00 (20.00 - 50.00) | 50.00 (25.50 - 65.00) | 67.00 (46.00 - 80.00) |
| **Community setting** | | | | | | | | | | |
|  | **Overall (n=122)** | **EU5 Overall (n=20)** | **USA**  **(n=61)** | **France (n=7)** | **Germany*** | **Italy*** | **Spain*** | **UK*** | **Japan (n=36)** | **China*** |
| **Proportion of patients tested in year prior to updated guidelines** | 20.00 (0.00 - 35.00) | 22.50 (18.75 - 32.50) | 15.00 (0.00 - 35.00) | 0.00 (0.00 - 17.50) |  |  |  |  | 10.00 (0.00 - 38.75) |  |
| **Proportion of patients tested in year after updated guidelines** | 50.00 (20.00 - 80.00) | 50.00 (23.75 - 72.50) | 50.00 (25.00 - 90.00) | 20.00 (8.00 - 35.00) |  |  |  |  | 40.00 (8.75 - 50.00) |  |
| **Secondary setting** | | | | | | | | | | |
|  | **Overall (n=22)** | **EU5 Overall (n=13)** | **USA*** | **France*** | **Germany*** | **Italy*** | **Spain*** | **UK*** | **Japan*** | **China*** |
| **Proportion of patients tested in year prior to updated guidelines** | 20.00 (5.00 - 50.00) | 20.00 (5.00 - 30.00) |  |  |  |  |  |  |  |  |
| **Proportion of patients tested in year after updated guidelines** | 40.00 (20.00 - 69.00) | 40.00 (20.00 - 66.00) |  |  |  |  |  |  |  |  |

*Results reported only for subgroups with >5 respondents. Key: EU5 – overall results from across France/Germany/Italy/Spain/UK, HRRm – homologous recombination repair mutation, mCRPC – metastatic castration-resistant prostate cancer, PARPi – poly-adenosine diphosphate-ribose polymerase inhibitor, United Kingdom, USA – United States.

**Supplementary Table 4. Physician reported frequency of recommending tissue biopsy/tumor testing, germline blood testing or ctDNA testing in the year prior to and following regulatory approval of PARP inhibitors (overall and by country)***

| **Frequency** | **Overall (n=407)** | **EU5 Overall (n=132)** | **USA (n=100)** | **France (n=27)** | **Germany (n=27)** | **Italy (n=28)** | **Spain (n=25)** | **UK**  **(n=25)** | **Japan (n=100)** | **China (n=75)** |
| --- | --- | --- | --- | --- | --- | --- | --- | --- | --- | --- |
| **Test type most frequently recommended prior to regulatory approval of PARPi, number of physicians * (%)** | | | | | | | | | | |
| **Tissue biopsy or tumour testing Pre** | 215 (52.83%) | 66 (50.00%) | 43 (43.00%) | 15 (55.56%) | 14 (51.85%) | 14 (50.00%) | 13 (52.00%) | 10 (40.00%) | 52 (52.00%) | 54 (72.00%) |
| **Germline blood testing pre** | 117 (28.75%) | 40 (30.30%) | 29 (29.00%) | 11 (40.74%) | 5 (18.52%) | 11 (39.29%) | 7 (28.00%) | 6 (24.00%) | 24 (24.00%) | 24 (32.00%) |
| **ctDNA testing** | 95 (23.34%) | 34 (25.76%) | 20 (20.00%) | 9 (33.33%) | 8 (29.63%) | 6 (21.43%) | 7 (28.00%) | 4 (16.00%) | 24 (24.00%) | 17 (22.67%) |
| **Test type most frequently recommended post-regulatory approval of PARP inhibitors, number of physicians * (%)** | | | | | | | | | | |
| **Tissue biopsy or tumour testing** | 265 (65.11%) | 83 (62.88%) | 58 (58.00%) | 18 (66.67%) | 16 (59.26%) | 17 (60.71%) | 17 (68.00%) | 15 (60.00%) | 61 (61.00%) | 63 (84.00%) |
| **Germline blood testing** | 156 (38.33%) | 64 (48.48%) | 37 (37.00%) | 13 (48.15%) | 14 (51.85%) | 15 (53.57%) | 10 (40.00%) | 12 (48.00%) | 26 (26.00%) | 29 (38.67%) |
|  |  |  |  |  |  |  |  |  |  |  |
| **ctDNA testing** | 130 (31.94%) | 50 (37.88%) | 27 (27.00%) | 13 (48.15%) | 12 (44.44%) | 10 (35.71%) | 9 (36.00%) | 6 (24.00%) | 25 (25.00%) | 28 (37.33%) |

*Respondents were asked indicate how frequently each test type was recommended before and after PARPi approval on a scale of 1–3 1= most frequent to 3= least frequent). Results for the % of physicians selecting each test type as number 1 / most frequently recommended test are presented here. Note that participants could specify the same number for more than one test and so results may not add up to 100%. Key: ctDNA – circulating tumor DNA, EU5 – overall results from across France/Germany/Italy/Spain/UK, HRRm - homologous recombination repair mutation, PARP - poly-adenosine diphosphate-ribose polymerase inhibitors, UK – United Kingdom, USA – United States

**Supplementary Table 5. The most common reasons/considerations for choosing tissue/germline blood/ctDNA testing as a second follow-up HRRm test***

| **Tissue test type** | **Overall** | **EU5** | **USA** | **France** | **Germany** | **Italy** | **Spain** | **UK** | **Japan** | **China** |
| --- | --- | --- | --- | --- | --- | --- | --- | --- | --- | --- |
| **Tissue** | **N (%=93)** | **N (%=30)** | **N (%=21)** | **N≤5** | **N (%=6)** | **N (%=11)** | **N≤5** | **N (%=7)** | **N (%=21)** | **N (%=21)** |
| A reliable diagnostic tool/reasonable alternative to retesting tissue samples | 1 (1.1%) | 1 (3.3%) | 0 (0%) | - | 0 (0%) | 1 (9.1%) | - | 0 (0%) | 0 (0%) | 0 (0%) |
| Availability and convenience of obtaining/accessing sample | 6 (6.5%) | 3 (10%) | 2 (9.5%) | - | 0 (0%) | 0 (0%) | - | 2 (28.6%) | 0 (0%) | 1 (4.8%) |
| Availability of laboratory resources | 1 (1.1%) | 1 (3.3%) | 0 (0%) | - | 0 (0%) | 0 (0%) | - | 0 (0%) | 0 (0%) | 0 (0%) |
| Depends on patient need | 3 (3.2%) | 0 (0%) | 1 (4.8%) | - | 0 (0%) | 0 (0%) | - | 0 (0%) | 0 (0%) | 2 (9.5%) |
| Following established guidelines/institution standard practice | 5 (5.4%) | 2 (6.7%) | 2 (9.5%) | - | 0 (0%) | 0 (0%) | - | 0 (0%) | 1 (4.8%) | 0 (0%) |
| Higher accuracy and reliability | 28 (30.1%) | 4 (13.3%) | 5 (23.8%) | - | 1 (16.7%) | 3 (27.3%) | - | 0 (0%) | 8 (38.1%) | 11 (52.4%) |
| Informs treatment decisions | 9 (9.7%) | 3 (10%) | 0 (0%) | - | 1 (16.7%) | 1 (9.1%) | - | 0 (0%) | 5 (23.8%) | 1 (4.8%) |
| More informative results (i.e. can identify other actionable mutations) | 16 (17.2%) | 9 (30%) | 0 (0%) | - | 3 (50%) | 2 (18.2%) | - | 4 (57.1%) | 4 (19%) | 3 (14.3%) |
| None | 7 (7.5%) | 0 (0%) | 2 (9.5%) | - | 0 (0%) | 0 (0%) | - | 0 (0%) | 5 (23.8%) | 0 (0%) |
| Other | 11 (11.8%) | 5 (16.7%) | 4 (19%) | - | 1 (16.7%) | 2 (18.2%) | - | 1 (14.3%) | 1 (4.8%) | 1 (4.8%) |
| Patient preference | 1 (1.1%) | 0 (0%) | 0 (0%) | - | 0 (0%) | 0 (0%) | - | 0 (0%) | 0 (0%) | 1 (4.8%) |
| Physician Judgement | 3 (3.2%) | 2 (6.7%) | 0 (0%) | - | 0 (0%) | 2 (18.2%) | - | 0 (0%) | 0 (0%) | 1 (4.8%) |
| Previous tests were not accurate | 2 (2.2%) | 1 (3.3%) | 1 (4.8%) | - | 0 (0%) | 0 (0%) | - | 1 (14.3%) | 0 (0%) | 0 (0%) |
| Quicker turnaround | 2 (2.2%) | 2 (6.7%) | 0 (0%) | - | 0 (0%) | 0 (0%) | - | 0 (0%) | 0 (0%) | 0 (0%) |
| Reduces patient burden/minimally invasive | 1 (1.1%) | 1 (3.3%) | 0 (0%) | - | 0 (0%) | 1 (9.1%) | - | 0 (0%) | 0 (0%) | 0 (0%) |
| Physician preference and familiarity | 2 (2.2%) | 0 (0%) | 2 (9.5%) | - | 0 (0%) | 0 (0%) | - | 0 (0%) | 0 (0%) | 0 (0%) |
| To assess genetic/genomic status | 2 (2.2%) | 0 (0%) | 2 (9.5%) | - | 0 (0%) | 0 (0%) | - | 0 (0%) | 0 (0%) | 0 (0%) |
| **Germline blood testing** | **N (%=164)** | **N (%=53)** | **N (%=38)** | **N (%=7)** | **N (%=13)** | **N (%=10)** | **N (%=15)** | **N (%=8)** | **N (%=28)** | **N (%=45)** |
| A reliable diagnostic tool/reasonable alternative to retesting tissue samples | 23 (14%) | 4 (7.5%) | 5 (13.2%) | 0 (0%) | 2 (15.4%) | 0 (0%) | 2 (13.3%) | 0 (0%) | 9 (20%) | 5 (17.9%) |
| Availability and convenience of obtaining/accessing sample | 50 (30.5%) | 14 (26.4%) | 9 (23.7%) | 2 (28.6%) | 3 (23.1%) | 3 (30%) | 5 (33.3%) | 1 (12.5%) | 13 (28.9%) | 14 (50%) |
| Common test | 1 (0.6%) | 1 (1.9%) | 0 (0%) | 0 (0%) | 0 (0%) | 0 (0%) | 1 (6.7%) | 0 (0%) | 0 (0%) | 0 (0%) |
| Depends on patient need | 1 (0.6%) | 0 (0%) | 0 (0%) | 0 (0%) | 0 (0%) | 0 (0%) | 0 (0%) | 0 (0%) | 0 (0%) | 1 (3.6%) |
| Following established guidelines/institution standard practice | 19 (11.6%) | 8 (15.1%) | 6 (15.8%) | 1 (14.3%) | 2 (15.4%) | 1 (10%) | 2 (13.3%) | 2 (25%) | 4 (8.9%) | 1 (3.6%) |
| Informs treatment decisions | 15 (9.1%) | 7 (13.2%) | 3 (7.9%) | 3 (42.9%) | 1 (7.7%) | 2 (20%) | 1 (6.7%) | 0 (0%) | 4 (8.9%) | 1 (3.6%) |
| None | 4 (2.4%) | 0 (0%) | 1 (2.6%) | 0 (0%) | 0 (0%) | 0 (0%) | 0 (0%) | 0 (0%) | 3 (6.7%) | 0 (0%) |
| Not implemented in practice yet | 2 (1.2%) | 1 (1.9%) | 0 (0%) | 0 (0%) | 1 (7.7%) | 0 (0%) | 0 (0%) | 0 (0%) | 1 (2.2%) | 0 (0%) |
| Other | 10 (6.1%) | 4 (7.5%) | 2 (5.3%) | 0 (0%) | 2 (15.4%) | 0 (0%) | 2 (13.3%) | 0 (0%) | 3 (6.7%) | 1 (3.6%) |
| Physician Judgement | 5 (3%) | 4 (7.5%) | 0 (0%) | 0 (0%) | 0 (0%) | 4 (40%) | 0 (0%) | 0 (0%) | 0 (0%) | 1 (3.6%) |
| Preferable next step after solid tumor testing. | 7 (4.3%) | 1 (1.9%) | 6 (15.8%) | 0 (0%) | 0 (0%) | 0 (0%) | 0 (0%) | 1 (12.5%) | 0 (0%) | 0 (0%) |
| Previous tests were not accurate | 1 (0.6%) | 0 (0%) | 0 (0%) | 0 (0%) | 0 (0%) | 0 (0%) | 0 (0%) | 0 (0%) | 0 (0%) | 1 (3.6%) |
| Quicker turnaround | 7 (4.3%) | 3 (5.7%) | 1 (2.6%) | 0 (0%) | 1 (7.7%) | 0 (0%) | 2 (13.3%) | 0 (0%) | 0 (0%) | 3 (10.7%) |
| To assess genetic/genomic status | 25 (15.2%) | 10 (18.9%) | 7 (18.4%) | 1 (14.3%) | 0 (0%) | 2 (20%) | 3 (20%) | 4 (50%) | 7 (15.6%) | 1 (3.6%) |
| Unavailable tissue samples | 7 (4.3%) | 2 (3.8%) | 3 (7.9%) | 0 (0%) | 2 (15.4%) | 0 (0%) | 0 (0%) | 0 (0%) | 1 (2.2%) | 1 (3.6%) |
| A reliable diagnostic tool | 3 (1.8%) | 0 (0%) | 3 (7.9%) | 0 (0%) | 0 (0%) | 0 (0%) | 0 (0%) | 0 (0%) | 0 (0%) | 0 (0%) |
| Lower cost/insurance and reimbursement coverage | 1 (0.6%) | 0 (0%) | 1 (2.6%) | 0 (0%) | 0 (0%) | 0 (0%) | 0 (0%) | 0 (0%) | 0 (0%) | 0 (0%) |
| **ctDNA testing** | **N (%=86)** | **N (%=27)** | **N (%=20)** | **N (%=9)** | **N≤5** | **N (%=6)** | **N (%=5)** | **N≤5** | **N (%=21)** | **N (%=18)** |
| A reliable diagnostic tool/reasonable alternative to retesting tissue samples | 2 (2.3%) | 2 (7.4%) | 0 (0%) | 1 (11.1%) | - | 1 (16.7%) | 0 (0%) | - | 0 (0%) | 0 (0%) |
| Availability and convenience of obtaining/accessing sample | 29 (33.7%) | 7 (25.9%) | 8 (40%) | 3 (33.3%) | - | 3 (50%) | 1 (20%) | - | 7 (33.3%) | 7 (38.9%) |
| Depends on patient need | 1 (1.2%) | 0 (0%) | 0 (0%) | 0 (0%) | - | 0 (0%) | 0 (0%) | - | 0 (0%) | 1 (5.6%) |
| Following established guidelines/institution standard practice | 6 (7%) | 2 (7.4%) | 1 (5%) | 0 (0%) | - | 0 (0%) | 0 (0%) | - | 3 (14.3%) | 0 (0%) |
| Higher accuracy and reliability | 21 (24.4%) | 9 (33.3%) | 2 (10%) | 1 (11.1%) | - | 1 (16.7%) | 2 (40%) | - | 4 (19%) | 6 (33.3%) |
| Informs treatment decisions | 7 (8.1%) | 2 (7.4%) | 0 (0%) | 1 (11.1%) | - | 1 (16.7%) | 0 (0%) | - | 1 (4.8%) | 4 (22.2%) |
| Lower cost/insurance and reimbursement coverage | 2 (2.3%) | 1 (3.7%) | 0 (0%) | 0 (0%) | - | 0 (0%) | 1 (20%) | - | 0 (0%) | 1 (5.6%) |
| More informative results (i.e. can identify other actionable mutations initial tumor) | 16 (18.6%) | 6 (22.2%) | 4 (20%) | 2 (22.2%) | - | 0 (0%) | 2 (40%) | - | 4 (19%) | 2 (11.1%) |
| Preferable next step after solid tumor testing. | 1 (1.2%) | 1 (3.7%) | 0 (0%) | 0 (0%) | - | 0 (0%) | 1 (20%) | - | 0 (0%) | 0 (0%) |
| Reduces patient burden/minimally invasive | 11 (12.8%) | 2 (7.4%) | 4 (20%) | 1 (11.1%) | - | 0 (0%) | 0 (0%) | - | 3 (14.3%) | 2 (11.1%) |
| To assess genetic/genomic status | 7 (8.1%) | 2 (7.4%) | 1 (5%) | 1 (11.1%) | - | 1 (16.7%) | 0 (0%) | - | 4 (19%) | 0 (0%) |
| Other | 1 (1.2%) | 0 (0%) | 1 (5%) | 0 (0%) | - | 0 (0%) | 0 (0%) | - | 0 (0%) | 0 (0%) |
| Physician preference and familiarity | 1 (1.2%) | 0 (0%) | 1 (5%) | 0 (0%) | - | 0 (0%) | 0 (0%) | - | 0 (0%) | 0 (0%) |

*Results reported only for subgroups with >5 respondents. Key: ctDNA – circulating tumor DNA, EU5 – overall results from across France/Germany/Italy/Spain/UK, HRRm - homologous recombination repair mutation, UK – United Kingdom, USA – United States.

**Supplementary Table 6: Most common characteristics associated with the decision to treat patients with mCRPC with PARPi NHAs and chemotherapy according to physicians (overall and by country)**

|  | **Overall^‡^** | **EU5** | **USA** | **France** | **Germany** | **Italy** | **Spain** | **UK** | **Japan** | **China** |
| --- | --- | --- | --- | --- | --- | --- | --- | --- | --- | --- |
| Oncologists and urologists: | **N (%=338)** | **N (%=107)** | **N (%=90)** | **N (%=22)** | **N (%=22)** | **N (%=23)** | **N (%=20)** | **N (%=20)** | **N (%=81)** | **N (%=60)** |
| **Characteristics associated with physician decision to treat mCRPC with PARPi*** | | | | | | | | | | |
| Age | 53 (15.68%) | 20 (18.69%) | 9 (10.00%) | 1 (4.55%) | 5 (22.73%) | 5 (21.74%) | 4 (20.00%) | 5 (25.00%) | 13 (16.05%) | 11 (18.33%) |
| ≤65 years | 19 (5.62%) | 8 (7.48%) | 2 (2.22%) | 0 (0.00%) | 2 (9.09%) | 2 (8.70%) | 2 (10.00%) | 2 (10.00%) | 7 (8.64%) | 2 (3.33%) |
| 66-79 years | 26 (7.69%) | 9 (8.41%) | 4 (4.44%) | 1 (4.55%) | 1 (4.55%) | 3 (13.04%) | 2 (10.00%) | 2 (10.00%) | 6 (7.41%) | 7 (11.67%) |
| ≥80 years | 8 (2.37%) | 3 (2.80%) | 3 (3.33%) | 0 (0.00%) | 2 (9.09%) | 0 (0.00%) | 0 (0.00%) | 1 (5.00%) | 0 (0.00%) | 2 (3.33%) |
| Fewer and less severe comorbidities | 72 (21.30%) | 11 (10.28%) | 17 (18.89%) | 1 (4.55%) | 2 (9.09%) | 1 (4.35%) | 1 (5.00%) | 6 (30.00%) | 20 (24.69%) | 24 (40.00%) |
| Toxicity profile of treatment (can tolerate adverse events) | 84 (24.85%) | 19 (17.76%) | 18 (20.00%) | 3 (13.64%) | 0 (0.00%) | 10 (43.48%) | 1 (5.00%) | 5 (25.00%) | 28 (34.57%) | 19 (31.67%) |
| Efficacy of treatment | 136 (40.24%) | 37 (34.58%) | 38 (42.22%) | 6 (27.27%) | 5 (22.73%) | 9 (39.13%) | 9 (45.00%) | 8 (40.00%) | 30 (37.04%) | 31 (51.67%) |
| HRR mutational status | 139 (41.12%) | 54 (50.47%) | 45 (50.00%) | 14 (63.64%) | 13 (59.09%) | 10 (43.48%) | 10 (50.00%) | 7 (35.00%) | 18 (22.22%) | 22 (36.67%) |
| Positive status | 136 (40.24%) | 54 (50.47%) | 45 (50.00%) | 14 (63.64%) | 13 (59.09%) | 10 (43.48%) | 10 (50.00%) | 7 (35.00%) | 17 (20.99%) | 20 (33.33%) |
| Negative status | 3 (0.89%) | 0 (0.00%) | 0 (0.00%) | 0 (0.00%) | 0 (0.00%) | 0 (0.00%) | 0 (0.00%) | 0 (0.00%) | 1 (1.23%) | 2 (3.33%) |
| Other biomarker mutational status | 51 (15.09%) | 24 (22.43%) | 7 (7.78%) | 5 (22.73%) | 10 (45.45%) | 3 (13.04%) | 3 (15.00%) | 3 (15.00%) | 7 (8.64%) | 13 (21.67%) |
| Positive status | 45 (13.31%) | 23 (21.50%) | 7 (7.78%) | 5 (22.73%) | 9 (40.91%) | 3 (13.04%) | 3 (15.00%) | 3 (15.00%) | 4 (4.94%) | 11 (18.33%) |
| Negative status | 6 (1.78%) | 1 (0.93%) | 0 (0.00%) | 0 (0.00%) | 1 (4.55%) | 0 (0.00%) | 0 (0.00%) | 0 (0.00%) | 3 (3.70%) | 2 (3.33%) |
| Lower cost of treatment | 30 (8.88%) | 3 (2.80%) | 5 (5.56%) | 0 (0.00%) | 0 (0.00%) | 1 (4.35%) | 1 (5.00%) | 1 (5.00%) | 7 (8.64%) | 15 (25.00%) |
| Ease of administration | 70 (20.71%) | 19 (17.76%) | 13 (14.44%) | 3 (13.64%) | 3 (13.64%) | 6 (26.09%) | 2 (10.00%) | 5 (25.00%) | 11 (13.58%) | 27 (45.00%) |
| Covered under reimbursement policy | 50 (14.79%) | 18 (16.82%) | 13 (14.44%) | 3 (13.64%) | 7 (31.82%) | 4 (17.39%) | 4 (20.00%) | 0 (0.00%) | 7 (8.64%) | 12 (20.00%) |
| ECOG performance status | 27 (7.99%) | 7 (6.54%) | 15 (16.67%) | 1 (4.55%) | 1 (4.55%) | 0 (0.00%) | 3 (15.00%) | 2 (10.00%) | 2 (2.47%) | 3 (5.00%) |
| ECOG PS 0-1 | 23 (6.80%) | 6 (5.61%) | 14 (15.56%) | 0 (0.00%) | 1 (4.55%) | 0 (0.00%) | 3 (15.00%) | 2 (10.00%) | 2 (2.47%) | 1 (1.67%) |
| ECOG PS 2-4 | 4 (1.18%) | 1 (0.93%) | 1 (1.11%) | 1 (4.55%) | 0 (0.00%) | 0 (0.00%) | 0 (0.00%) | 0 (0.00%) | 0 (0.00%) | 2 (3.33%) |
| Patient choice | 54 (15.98%) | 14 (13.08%) | 11 (12.22%) | 1 (4.55%) | 3 (13.64%) | 3 (13.04%) | 3 (15.00%) | 4 (20.00%) | 9 (11.11%) | 20 (33.33%) |
| High Gleason score | 42 (12.43%) | 14 (13.08%) | 12 (13.33%) | 1 (4.55%) | 3 (13.64%) | 5 (21.74%) | 3 (15.00%) | 2 (10.00%) | 7 (8.64%) | 9 (15.00%) |
| High baseline PSA level | 41 (12.13%) | 8 (7.48%) | 12 (13.33%) | 0 (0.00%) | 1 (4.55%) | 3 (13.04%) | 2 (10.00%) | 2 (10.00%) | 8 (9.88%) | 13 (21.67%) |
| Shorter PSADT | 22 (6.51%) | 5 (4.67%) | 8 (8.89%) | 1 (4.55%) | 1 (4.55%) | 2 (8.70%) | 0 (0.00%) | 1 (5.00%) | 5 (6.17%) | 4 (6.67%) |
| ≤3 months | 8 (2.37%) | 3 (2.80%) | 2 (2.22%) | 1 (4.55%) | 0 (0.00%) | 1 (4.35%) | 0 (0.00%) | 1 (5.00%) | 2 (2.47%) | 1 (1.67%) |
| ≤6 months | 13 (3.85%) | 2 (1.87%) | 6 (6.67%) | 0 (0.00%) | 1 (4.55%) | 1 (4.35%) | 0 (0.00%) | 0 (0.00%) | 3 (3.70%) | 2 (3.33%) |
| >6 months | 1 (0.30%) | 0 (0.00%) | 0 (0.00%) | 0 (0.00%) | 0 (0.00%) | 0 (0.00%) | 0 (0.00%) | 0 (0.00%) | 0 (0.00%) | 1 (1.67%) |
| Prior treatment | 58 (17.16%) | 19 (17.76%) | 17 (18.89%) | 2 (9.09%) | 5 (22.73%) | 5 (21.74%) | 5 (25.00%) | 2 (10.00%) | 10 (12.35%) | 12 (20.00%) |
| High volume disease | 38 (11.24%) | 14 (13.08%) | 8 (8.89%) | 1 (4.55%) | 2 (9.09%) | 4 (17.39%) | 3 (15.00%) | 4 (20.00%) | 5 (6.17%) | 11 (18.33%) |
| Metastases of organ | 35 (10.36%) | 8 (7.48%) | 7 (7.78%) | 0 (0.00%) | 1 (4.55%) | 3 (13.04%) | 2 (10.00%) | 2 (10.00%) | 9 (11.11%) | 11 (18.33%) |
| Guidelines | 65 (19.23%) | 27 (25.23%) | 17 (18.89%) | 7 (31.82%) | 9 (40.91%) | 5 (21.74%) | 3 (15.00%) | 3 (15.00%) | 7 (8.64%) | 14 (23.33%) |
| Other | 8 (2.37%) | 1 (0.93%) | 6 (6.67%) | 0 (0.00%) | 0 (0.00%) | 0 (0.00%) | 0 (0.00%) | 1 (5.00%) | 1 (1.23%) | 0 (0.00%) |
| **Characteristics associated with physician decision to treat mCRPC with NHA*** | | | | | | | | | | |
| Age | 92 (27.22%) | 34 (31.78%) | 24 (26.67%) | 7 (31.82%) | 7 (31.82%) | 6 (26.09%) | 5 (25.00%) | 9 (45.00%) | 22 (27.16%) | 12 (20.00%) |
| ≤65 years | 25 (7.40%) | 6 (5.61%) | 4 (4.44%) | 1 (4.55%) | 0 (0.00%) | 1 (4.35%) | 2 (10.00%) | 2 (10.00%) | 9 (11.11%) | 6 (10.00%) |
| 66-79 years | 40 (11.83%) | 12 (11.21%) | 14 (15.56%) | 1 (4.55%) | 3 (13.64%) | 3 (13.04%) | 3 (15.00%) | 2 (10.00%) | 9 (11.11%) | 5 (8.33%) |
| ≥80 years | 27 (7.99%) | 16 (14.95%) | 6 (6.67%) | 5 (22.73%) | 4 (18.18%) | 2 (8.70%) | 0 (0.00%) | 5 (25.00%) | 4 (4.94%) | 1 (1.67%) |
| Fewer and less severe comorbidities | 139 (41.12%) | 40 (37.38%) | 30 (33.33%) | 3 (13.64%) | 11 (50.00%) | 10 (43.48%) | 5 (25.00%) | 11 (55.00%) | 35 (43.21%) | 34 (56.67%) |
| Toxicity profile of treatment (can tolerate adverse events) | 127 (37.57%) | 46 (42.99%) | 25 (27.78%) | 11 (50.00%) | 6 (27.27%) | 11 (47.83%) | 9 (45.00%) | 9 (45.00%) | 34 (41.98%) | 22 (36.67%) |
| Efficacy of treatment | 191 (56.51%) | 59 (55.14%) | 64 (71.11%) | 13 (59.09%) | 11 (50.00%) | 12 (52.17%) | 14 (70.00%) | 9 (45.00%) | 26 (32.10%) | 42 (70.00%) |
| HRR mutational status | 48 (14.20%) | 16 (14.95%) | 13 (14.44%) | 0 (0.00%) | 5 (22.73%) | 6 (26.09%) | 4 (20.00%) | 1 (5.00%) | 7 (8.64%) | 12 (20.00%) |
| Positive status | 36 (10.65%) | 10 (9.35%) | 10 (11.11%) | 0 (0.00%) | 2 (9.09%) | 5 (21.74%) | 2 (10.00%) | 1 (5.00%) | 6 (7.41%) | 10 (16.67%) |
| Negative status | 12 (3.55%) | 6 (5.61%) | 3 (3.33%) | 0 (0.00%) | 3 (13.64%) | 1 (4.35%) | 2 (10.00%) | 0 (0.00%) | 1 (1.23%) | 2 (3.33%) |
| Other biomarker mutational status | 21 (6.21%) | 9 (8.41%) | 2 (2.22%) | 1 (4.55%) | 2 (9.09%) | 4 (17.39%) | 2 (10.00%) | 0 (0.00%) | 5 (6.17%) | 5 (8.33%) |
| Positive status | 16 (4.73%) | 8 (7.48%) | 1 (1.11%) | 1 (4.55%) | 2 (9.09%) | 4 (17.39%) | 1 (5.00%) | 0 (0.00%) | 3 (3.70%) | 4 (6.67%) |
| Negative status | 5 (1.48%) | 1 (0.93%) | 1 (1.11%) | 0 (0.00%) | 0 (0.00%) | 0 (0.00%) | 1 (5.00%) | 0 (0.00%) | 2 (2.47%) | 1 (1.67%) |
| Lower cost of treatment | 40 (11.83%) | 10 (9.35%) | 11 (12.22%) | 0 (0.00%) | 3 (13.64%) | 3 (13.04%) | 2 (10.00%) | 2 (10.00%) | 9 (11.11%) | 10 (16.67%) |
| Ease of administration | 127 (37.57%) | 46 (42.99%) | 41 (45.56%) | 11 (50.00%) | 11 (50.00%) | 8 (34.78%) | 10 (50.00%) | 6 (30.00%) | 16 (19.75%) | 24 (40.00%) |
| Covered under reimbursement policy | 60 (17.75%) | 23 (21.50%) | 15 (16.67%) | 2 (9.09%) | 8 (36.36%) | 6 (26.09%) | 7 (35.00%) | 0 (0.00%) | 4 (4.94%) | 18 (30.00%) |
| ECOG performance status | 41 (12.13%) | 16 (14.95%) | 21 (23.33%) | 3 (13.64%) | 4 (18.18%) | 2 (8.70%) | 3 (15.00%) | 4 (20.00%) | 0 (0.00%) | 4 (6.67%) |
| ECOG PS 0-1 | 25 (7.40%) | 8 (7.48%) | 13 (14.44%) | 1 (4.55%) | 1 (4.55%) | 0 (0.00%) | 2 (10.00%) | 4 (20.00%) | 0 (0.00%) | 4 (6.67%) |
| ECOG PS 2-4 | 16 (4.73%) | 8 (7.48%) | 8 (8.89%) | 2 (9.09%) | 3 (13.64%) | 2 (8.70%) | 1 (5.00%) | 0 (0.00%) | 0 (0.00%) | 0 (0.00%) |
| Patient choice | 75 (22.19%) | 25 (23.36%) | 21 (23.33%) | 2 (9.09%) | 7 (31.82%) | 4 (17.39%) | 6 (30.00%) | 6 (30.00%) | 15 (18.52%) | 14 (23.33%) |
| High Gleason score | 52 (15.38%) | 14 (13.08%) | 14 (15.56%) | 2 (9.09%) | 4 (18.18%) | 5 (21.74%) | 2 (10.00%) | 1 (5.00%) | 8 (9.88%) | 16 (26.67%) |
| High baseline PSA level | 39 (11.54%) | 10 (9.35%) | 9 (10.00%) | 0 (0.00%) | 1 (4.55%) | 5 (21.74%) | 0 (0.00%) | 4 (20.00%) | 10 (12.35%) | 10 (16.67%) |
| Shorter PSADT | 19 (5.62%) | 9 (8.41%) | 5 (5.56%) | 1 (4.55%) | 1 (4.55%) | 3 (13.04%) | 1 (5.00%) | 3 (15.00%) | 2 (2.47%) | 3 (5.00%) |
| ≤3 months | 5 (1.48%) | 1 (0.93%) | 1 (1.11%) | 0 (0.00%) | 0 (0.00%) | 1 (4.35%) | 0 (0.00%) | 0 (0.00%) | 1 (1.23%) | 2 (3.33%) |
| ≤6 months | 10 (2.96%) | 6 (5.61%) | 3 (3.33%) | 1 (4.55%) | 1 (4.55%) | 2 (8.70%) | 0 (0.00%) | 2 (10.00%) | 1 (1.23%) | 0 (0.00%) |
| >6 months | 4 (1.18%) | 2 (1.87%) | 1 (1.11%) | 0 (0.00%) | 0 (0.00%) | 0 (0.00%) | 1 (5.00%) | 1 (5.00%) | 0 (0.00%) | 1 (1.67%) |
| Prior treatment | 65 (19.23%) | 25 (23.36%) | 20 (22.22%) | 4 (18.18%) | 7 (31.82%) | 7 (30.43%) | 3 (15.00%) | 4 (20.00%) | 11 (13.58%) | 9 (15.00%) |
| High volume disease | 35 (10.36%) | 14 (13.08%) | 9 (10.00%) | 1 (4.55%) | 1 (4.55%) | 6 (26.09%) | 3 (15.00%) | 3 (15.00%) | 0 (0.00%) | 12 (20.00%) |
| Metastases of organ | 28 (8.28%) | 8 (7.48%) | 10 (11.11%) | 0 (0.00%) | 1 (4.55%) | 4 (17.39%) | 1 (5.00%) | 2 (10.00%) | 2 (2.47%) | 8 (13.33%) |
| Guidelines | 67 (19.82%) | 25 (23.36%) | 27 (30.00%) | 8 (36.36%) | 7 (31.82%) | 6 (26.09%) | 0 (0.00%) | 4 (20.00%) | 4 (4.94%) | 11 (18.33%) |
| Other | 5 (1.48%) | 2 (1.87%) | 2 (2.22%) | 1 (4.55%) | 0 (0.00%) | 0 (0.00%) | 0 (0.00%) | 1 (5.00%) | 1 (1.23%) | 0 (0.00%) |
| **Characteristics associated with physician decision to treat mCRPC with chemotherapy (docetaxel)*** | | | | | | | | | | |
| Age | 129 (38.17%) | 47 (43.93%) | 32 (35.56%) | 9 (40.91%) | 10 (45.45%) | 9 (39.13%) | 10 (50.00%) | 9 (45.00%) | 29 (35.80%) | 21 (35.00%) |
| ≤65 years | 80 (23.67%) | 30 (28.04%) | 19 (21.11%) | 6 (27.27%) | 4 (18.18%) | 5 (21.74%) | 8 (40.00%) | 7 (35.00%) | 17 (20.99%) | 14 (23.33%) |
| 66-79 years | 35 (10.36%) | 8 (7.48%) | 11 (12.22%) | 1 (4.55%) | 3 (13.64%) | 1 (4.35%) | 1 (5.00%) | 2 (10.00%) | 11 (13.58%) | 5 (8.33%) |
| ≥80 years | 14 (4.14%) | 9 (8.41%) | 2 (2.22%) | 2 (9.09%) | 3 (13.64%) | 3 (13.04%) | 1 (5.00%) | 0 (0.00%) | 1 (1.23%) | 2 (3.33%) |
| Fewer and less severe comorbidities | 132 (39.05%) | 47 (43.93%) | 22 (24.44%) | 5 (22.73%) | 11 (50.00%) | 11 (47.83%) | 8 (40.00%) | 12 (60.00%) | 31 (38.27%) | 32 (53.33%) |
| Toxicity profile of treatment (can tolerate adverse events) | 137 (40.53%) | 40 (37.38%) | 31 (34.44%) | 5 (22.73%) | 9 (40.91%) | 7 (30.43%) | 7 (35.00%) | 12 (60.00%) | 36 (44.44%) | 30 (50.00%) |
| Efficacy of treatment | 157 (46.45%) | 45 (42.06%) | 49 (54.44%) | 9 (40.91%) | 7 (31.82%) | 13 (56.52%) | 9 (45.00%) | 7 (35.00%) | 30 (37.04%) | 33 (55.00%) |
| HRR mutational status | 41 (12.13%) | 12 (11.21%) | 11 (12.22%) | 1 (4.55%) | 4 (18.18%) | 4 (17.39%) | 3 (15.00%) | 0 (0.00%) | 7 (8.64%) | 11 (18.33%) |
| Positive status | 19 (5.62%) | 7 (6.54%) | 3 (3.33%) | 0 (0.00%) | 2 (9.09%) | 3 (13.04%) | 2 (10.00%) | 0 (0.00%) | 4 (4.94%) | 5 (8.33%) |
| Negative status | 22 (6.51%) | 5 (4.67%) | 8 (8.89%) | 1 (4.55%) | 2 (9.09%) | 1 (4.35%) | 1 (5.00%) | 0 (0.00%) | 3 (3.70%) | 6 (10.00%) |
| Other biomarker mutational status | 20 (5.92%) | 9 (8.41%) | 3 (3.33%) | 2 (9.09%) | 1 (4.55%) | 5 (21.74%) | 1 (5.00%) | 0 (0.00%) | 4 (4.94%) | 4 (6.67%) |
| Positive status | 14 (4.14%) | 6 (5.61%) | 3 (3.33%) | 0 (0.00%) | 0 (0.00%) | 5 (21.74%) | 1 (5.00%) | 0 (0.00%) | 2 (2.47%) | 3 (5.00%) |
| Negative status | 6 (1.78%) | 3 (2.80%) | 0 (0.00%) | 2 (9.09%) | 1 (4.55%) | 0 (0.00%) | 0 (0.00%) | 0 (0.00%) | 2 (2.47%) | 1 (1.67%) |
| Lower cost of treatment | 71 (21.01%) | 15 (14.02%) | 13 (14.44%) | 0 (0.00%) | 5 (22.73%) | 3 (13.04%) | 2 (10.00%) | 5 (25.00%) | 7 (8.64%) | 36 (60.00%) |
| Ease of administration | 66 (19.53%) | 19 (17.76%) | 12 (13.33%) | 1 (4.55%) | 3 (13.64%) | 8 (34.78%) | 2 (10.00%) | 5 (25.00%) | 8 (9.88%) | 27 (45.00%) |
| Covered under reimbursement policy | 57 (16.86%) | 12 (11.21%) | 16 (17.78%) | 1 (4.55%) | 3 (13.64%) | 4 (17.39%) | 3 (15.00%) | 1 (5.00%) | 8 (9.88%) | 21 (35.00%) |
| ECOG performance status | 76 (22.49%) | 30 (28.04%) | 28 (31.11%) | 5 (22.73%) | 5 (22.73%) | 3 (13.04%) | 7 (35.00%) | 10 (50.00%) | 8 (9.88%) | 10 (16.67%) |
| ECOG PS 0-1 | 73 (21.60%) | 28 (26.17%) | 28 (31.11%) | 5 (22.73%) | 4 (18.18%) | 3 (13.04%) | 7 (35.00%) | 9 (45.00%) | 7 (8.64%) | 10 (16.67%) |
| ECOG PS 2-4 | 3 (0.89%) | 2 (1.87%) | 0 (0.00%) | 0 (0.00%) | 1 (4.55%) | 0 (0.00%) | 0 (0.00%) | 1 (5.00%) | 1 (1.23%) | 0 (0.00%) |
| Patient choice | 65 (19.23%) | 17 (15.89%) | 14 (15.56%) | 0 (0.00%) | 4 (18.18%) | 2 (8.70%) | 5 (25.00%) | 6 (30.00%) | 16 (19.75%) | 18 (30.00%) |
| High Gleason score | 89 (26.33%) | 31 (28.97%) | 25 (27.78%) | 8 (36.36%) | 6 (27.27%) | 10 (43.48%) | 4 (20.00%) | 3 (15.00%) | 20 (24.69%) | 13 (21.67%) |
| High baseline PSA level | 62 (18.34%) | 25 (23.36%) | 11 (12.22%) | 2 (9.09%) | 4 (18.18%) | 12 (52.17%) | 4 (20.00%) | 3 (15.00%) | 16 (19.75%) | 10 (16.67%) |
| Shorter PSADT | 42 (12.43%) | 15 (14.02%) | 18 (20.00%) | 4 (18.18%) | 4 (18.18%) | 3 (13.04%) | 1 (5.00%) | 3 (15.00%) | 8 (9.88%) | 1 (1.67%) |
| ≤3 months | 13 (3.85%) | 6 (5.61%) | 6 (6.67%) | 3 (13.64%) | 1 (4.55%) | 0 (0.00%) | 1 (5.00%) | 1 (5.00%) | 1 (1.23%) | 0 (0.00%) |
| ≤6 months | 27 (7.99%) | 9 (8.41%) | 12 (13.33%) | 1 (4.55%) | 3 (13.64%) | 3 (13.04%) | 0 (0.00%) | 2 (10.00%) | 6 (7.41%) | 0 (0.00%) |
| >6 months | 2 (0.59%) | 0 (0.00%) | 0 (0.00%) | 0 (0.00%) | 0 (0.00%) | 0 (0.00%) | 0 (0.00%) | 0 (0.00%) | 1 (1.23%) | 1 (1.67%) |
| Prior treatment | 73 (21.60%) | 28 (26.17%) | 21 (23.33%) | 3 (13.64%) | 7 (31.82%) | 11 (47.83%) | 5 (25.00%) | 2 (10.00%) | 13 (16.05%) | 11 (18.33%) |
| High volume disease | 99 (29.29%) | 46 (42.99%) | 31 (34.44%) | 9 (40.91%) | 10 (45.45%) | 9 (39.13%) | 11 (55.00%) | 7 (35.00%) | 5 (6.17%) | 17 (28.33%) |
| Metastases of organ | 94 (27.81%) | 32 (29.91%) | 32 (35.56%) | 8 (36.36%) | 5 (22.73%) | 7 (30.43%) | 9 (45.00%) | 3 (15.00%) | 17 (20.99%) | 13 (21.67%) |
| Guidelines | 49 (14.50%) | 22 (20.56%) | 14 (15.56%) | 9 (40.91%) | 5 (22.73%) | 4 (17.39%) | 0 (0.00%) | 4 (20.00%) | 4 (4.94%) | 9 (15.00%) |
| Other | 8 (2.37%) | 2 (1.87%) | 5 (5.56%) | 1 (4.55%) | 0 (0.00%) | 0 (0.00%) | 1 (5.00%) | 0 (0.00%) | 1 (1.23%) | 0 (0.00%) |
| *Responses not mutually exclusive; respondents selected all options that applied. Key: ECOG – Eastern Cooperative Oncology Group, EU5 – overall results from across France/Germany/Italy/Spain/UK, Physicians – healthcare providers, HRRm – homologous recombination repair genes, mCRPC – metastatic castration-resistant prostate cancer, NHA- next-generation hormonal agents, PARPi- poly-adenosine diphosphate-ribose polymerase inhibitors, PS – performance status, PSA – prostate-specific antigen, PSADT – PSA doubling time. | | | | | | | | | | |

**Supplementary Table 7. Physician reported patient characteristics associated with the decision to treat nmCRPC patients (overall and by country)**

|  | **Overall** | **EU5** | **USA** | **France** | **Germany** | **Italy** | **Spain** | **UK** | **Japan** | **China** |
| --- | --- | --- | --- | --- | --- | --- | --- | --- | --- | --- |
| Oncologists and urologists: | **N (%=338)** | **N (%=107)** | **N (%=90)** | **N (%=22)** | **N (%=22)** | **N (%=23)** | **N (%=20)** | **N (%=20)** | **N (%=81)** | **N (%=60)** |
| **Characteristics associated with the decision to treat nmCRPC patients with ADT + NHAs** | | | | | | | | | | |
| Age | 52 (15.38%) | 17 (15.89%) | 16 (17.78%) | 2 (9.09%) | 3 (13.64%) | 4 (17.39%) | 3 (15.00%) | 5 (25.00%) | 11 (13.58%) | 8 (13.33%) |
| ≤65 years | 24 (7.10%) | 8 (7.48%) | 4 (4.44%) | 1 (4.55%) | 1 (4.55%) | 2 (8.70%) | 3 (15.00%) | 1 (5.00%) | 8 (9.88%) | 4 (6.67%) |
| 66-79 years | 21 (6.21%) | 7 (6.54%) | 10 (11.11%) | 0 (0.00%) | 2 (9.09%) | 2 (8.70%) | 0 (0.00%) | 3 (15.00%) | 1 (1.23%) | 3 (5.00%) |
| ≥80 years | 7 (2.07%) | 2 (1.87%) | 2 (2.22%) | 1 (4.55%) | 0 (0.00%) | 0 (0.00%) | 0 (0.00%) | 1 (5.00%) | 2 (2.47%) | 1 (1.67%) |
| Fewer and less severe comorbidities | 102 (30.18%) | 18 (16.82%) | 26 (28.89%) | 2 (9.09%) | 3 (13.64%) | 3 (13.04%) | 2 (10.00%) | 8 (40.00%) | 25 (30.86%) | 33 (55.00%) |
| Toxicity profile of treatment (can tolerate adverse events) | 100 (29.59%) | 30 (28.04%) | 27 (30.00%) | 4 (18.18%) | 9 (40.91%) | 4 (17.39%) | 6 (30.00%) | 7 (35.00%) | 17 (20.99%) | 26 (43.33%) |
| Efficacy of treatment | 191 (56.51%) | 63 (58.88%) | 57 (63.33%) | 11 (50.00%) | 14 (63.64%) | 12 (52.17%) | 13 (65.00%) | 13 (65.00%) | 35 (43.21%) | 36 (60.00%) |
| Lower cost of treatment | 37 (10.95%) | 3 (2.80%) | 12 (13.33%) | 1 (4.55%) | 1 (4.55%) | 1 (4.35%) | 0 (0.00%) | 0 (0.00%) | 12 (14.81%) | 10 (16.67%) |
| Ease of administration | 103 (30.47%) | 39 (36.45%) | 28 (31.11%) | 9 (40.91%) | 8 (36.36%) | 8 (34.78%) | 8 (40.00%) | 6 (30.00%) | 14 (17.28%) | 22 (36.67%) |
| Covered under reimbursement policy | 64 (18.93%) | 24 (22.43%) | 18 (20.00%) | 5 (22.73%) | 7 (31.82%) | 5 (21.74%) | 6 (30.00%) | 1 (5.00%) | 8 (9.88%) | 14 (23.33%) |
| ECOG performance status | 35 (10.36%) | 17 (15.89%) | 11 (12.22%) | 2 (9.09%) | 2 (9.09%) | 2 (8.70%) | 2 (10.00%) | 9 (45.00%) | 2 (2.47%) | 5 (8.33%) |
| ECOG PS 0-1 | 29 (8.58%) | 14 (13.08%) | 9 (10.00%) | 1 (4.55%) | 1 (4.55%) | 1 (4.35%) | 2 (10.00%) | 9 (45.00%) | 2 (2.47%) | 4 (6.67%) |
| ECOG PS 2-4 | 6 (1.78%) | 3 (2.80%) | 2 (2.22%) | 1 (4.55%) | 1 (4.55%) | 1 (4.35%) | 0 (0.00%) | 0 (0.00%) | 0 (0.00%) | 1 (1.67%) |
| Patient choice | 66 (19.53%) | 15 (14.02%) | 19 (21.11%) | 1 (4.55%) | 1 (4.55%) | 4 (17.39%) | 1 (5.00%) | 8 (40.00%) | 9 (11.11%) | 23 (38.33%) |
| High Gleason score | 67 (19.82%) | 19 (17.76%) | 18 (20.00%) | 3 (13.64%) | 6 (27.27%) | 3 (13.04%) | 3 (15.00%) | 4 (20.00%) | 12 (14.81%) | 18 (30.00%) |
| High baseline PSA level | 51 (15.09%) | 11 (10.28%) | 11 (12.22%) | 1 (4.55%) | 2 (9.09%) | 3 (13.04%) | 2 (10.00%) | 3 (15.00%) | 15 (18.52%) | 14 (23.33%) |
| Shorter PSADT | 63 (18.64%) | 25 (23.36%) | 18 (20.00%) | 5 (22.73%) | 5 (22.73%) | 4 (17.39%) | 5 (25.00%) | 6 (30.00%) | 12 (14.81%) | 8 (13.33%) |
| ≤3 months | 19 (5.62%) | 7 (6.54%) | 2 (2.22%) | 1 (4.55%) | 0 (0.00%) | 3 (13.04%) | 1 (5.00%) | 2 (10.00%) | 3 (3.70%) | 7 (11.67%) |
| ≤6 months | 42 (12.43%) | 17 (15.89%) | 16 (17.78%) | 4 (18.18%) | 4 (18.18%) | 1 (4.35%) | 4 (20.00%) | 4 (20.00%) | 8 (9.88%) | 1 (1.67%) |
| >6 months | 2 (0.59%) | 1 (0.93%) | 0 (0.00%) | 0 (0.00%) | 1 (4.55%) | 0 (0.00%) | 0 (0.00%) | 0 (0.00%) | 1 (1.23%) | 0 (0.00%) |
| Prior treatment | 63 (18.64%) | 20 (18.69%) | 15 (16.67%) | 4 (18.18%) | 5 (22.73%) | 5 (21.74%) | 4 (20.00%) | 2 (10.00%) | 13 (16.05%) | 15 (25.00%) |
| High volume disease | 38 (11.24%) | 8 (7.48%) | 9 (10.00%) | 0 (0.00%) | 0 (0.00%) | 3 (13.04%) | 2 (10.00%) | 3 (15.00%) | 5 (6.17%) | 16 (26.67%) |
| Metastases of organ | 35 (10.36%) | 7 (6.54%) | 10 (11.11%) | 0 (0.00%) | 1 (4.55%) | 2 (8.70%) | 1 (5.00%) | 3 (15.00%) | 7 (8.64%) | 11 (18.33%) |
| Guidelines | 77 (22.78%) | 29 (27.10%) | 28 (31.11%) | 9 (40.91%) | 8 (36.36%) | 7 (30.43%) | 1 (5.00%) | 4 (20.00%) | 6 (7.41%) | 14 (23.33%) |
| Other | 3 (0.89%) | 0 (0.00%) | 2 (2.22%) | 0 (0.00%) | 0 (0.00%) | 0 (0.00%) | 0 (0.00%) | 0 (0.00%) | 1 (1.23%) | 0 (0.00%) |
| **Characteristics associated with the decision to treat nmCRPC patients with ADT + another hormone therapy** | | | | | | | | | | |
| Age | 65 (19.23%) | 22 (20.56%) | 14 (15.56%) | 5 (22.73%) | 5 (22.73%) | 5 (21.74%) | 3 (15.00%) | 4 (20.00%) | 19 (23.46%) | 10 (16.67%) |
| ≤65 years | 25 (7.40%) | 9 (8.41%) | 5 (5.56%) | 2 (9.09%) | 1 (4.55%) | 2 (8.70%) | 3 (15.00%) | 1 (5.00%) | 6 (7.41%) | 5 (8.33%) |
| 66-79 years | 25 (7.40%) | 8 (7.48%) | 5 (5.56%) | 3 (13.64%) | 2 (9.09%) | 3 (13.04%) | 0 (0.00%) | 0 (0.00%) | 9 (11.11%) | 3 (5.00%) |
| ≥80 years | 15 (4.44%) | 5 (4.67%) | 4 (4.44%) | 0 (0.00%) | 2 (9.09%) | 0 (0.00%) | 0 (0.00%) | 3 (15.00%) | 4 (4.94%) | 2 (3.33%) |
| Fewer and less severe comorbidities | 96 (28.40%) | 21 (19.63%) | 27 (30.00%) | 2 (9.09%) | 3 (13.64%) | 6 (26.09%) | 4 (20.00%) | 6 (30.00%) | 21 (25.93%) | 27 (45.00%) |
| Toxicity profile of treatment (can tolerate adverse events) | 109 (32.25%) | 29 (27.10%) | 36 (40.00%) | 4 (18.18%) | 8 (36.36%) | 3 (13.04%) | 7 (35.00%) | 7 (35.00%) | 20 (24.69%) | 24 (40.00%) |
| Efficacy of treatment | 155 (45.86%) | 54 (50.47%) | 41 (45.56%) | 12 (54.55%) | 10 (45.45%) | 12 (52.17%) | 11 (55.00%) | 9 (45.00%) | 28 (34.57%) | 32 (53.33%) |
| Lower cost of treatment | 64 (18.93%) | 13 (12.15%) | 14 (15.56%) | 2 (9.09%) | 1 (4.55%) | 3 (13.04%) | 2 (10.00%) | 5 (25.00%) | 16 (19.75%) | 21 (35.00%) |
| Ease of administration | 93 (27.51%) | 29 (27.10%) | 25 (27.78%) | 7 (31.82%) | 6 (27.27%) | 3 (13.04%) | 6 (30.00%) | 7 (35.00%) | 17 (20.99%) | 22 (36.67%) |
| Covered under reimbursement policy | 69 (20.41%) | 18 (16.82%) | 20 (22.22%) | 2 (9.09%) | 4 (18.18%) | 5 (21.74%) | 6 (30.00%) | 1 (5.00%) | 8 (9.88%) | 23 (38.33%) |
| ECOG performance status | 31 (9.17%) | 12 (11.21%) | 13 (14.44%) | 1 (4.55%) | 1 (4.55%) | 2 (8.70%) | 3 (15.00%) | 5 (25.00%) | 4 (4.94%) | 2 (3.33%) |
| ECOG PS 0-1 | 25 (7.40%) | 8 (7.48%) | 11 (12.22%) | 1 (4.55%) | 0 (0.00%) | 1 (4.35%) | 2 (10.00%) | 4 (20.00%) | 4 (4.94%) | 2 (3.33%) |
| ECOG PS 2-4 | 6 (1.78%) | 4 (3.74%) | 2 (2.22%) | 0 (0.00%) | 1 (4.55%) | 1 (4.35%) | 1 (5.00%) | 1 (5.00%) | 0 (0.00%) | 0 (0.00%) |
| Patient choice | 64 (18.93%) | 15 (14.02%) | 17 (18.89%) | 2 (9.09%) | 4 (18.18%) | 3 (13.04%) | 2 (10.00%) | 4 (20.00%) | 11 (13.58%) | 21 (35.00%) |
| High Gleason score | 63 (18.64%) | 21 (19.63%) | 16 (17.78%) | 1 (4.55%) | 7 (31.82%) | 7 (30.43%) | 3 (15.00%) | 3 (15.00%) | 10 (12.35%) | 16 (26.67%) |
| High baseline PSA level | 42 (12.43%) | 11 (10.28%) | 9 (10.00%) | 2 (9.09%) | 2 (9.09%) | 5 (21.74%) | 0 (0.00%) | 2 (10.00%) | 7 (8.64%) | 15 (25.00%) |
| Shorter PSADT | 67 (19.82%) | 24 (22.43%) | 18 (20.00%) | 3 (13.64%) | 4 (18.18%) | 5 (21.74%) | 5 (25.00%) | 7 (35.00%) | 15 (18.52%) | 10 (16.67%) |
| ≤3 months | 21 (6.21%) | 8 (7.48%) | 4 (4.44%) | 1 (4.55%) | 1 (4.55%) | 2 (8.70%) | 1 (5.00%) | 3 (15.00%) | 3 (3.70%) | 6 (10.00%) |
| ≤6 months | 40 (11.83%) | 14 (13.08%) | 13 (14.44%) | 2 (9.09%) | 2 (9.09%) | 3 (13.04%) | 4 (20.00%) | 3 (15.00%) | 9 (11.11%) | 4 (6.67%) |
| >6 months | 6 (1.78%) | 2 (1.87%) | 1 (1.11%) | 0 (0.00%) | 1 (4.55%) | 0 (0.00%) | 0 (0.00%) | 1 (5.00%) | 3 (3.70%) | 0 (0.00%) |
| Prior treatment | 56 (16.57%) | 18 (16.82%) | 16 (17.78%) | 3 (13.64%) | 3 (13.64%) | 5 (21.74%) | 3 (15.00%) | 4 (20.00%) | 8 (9.88%) | 14 (23.33%) |
| High volume disease | 37 (10.95%) | 8 (7.48%) | 11 (12.22%) | 1 (4.55%) | 0 (0.00%) | 2 (8.70%) | 4 (20.00%) | 1 (5.00%) | 4 (4.94%) | 14 (23.33%) |
| Metastases of organ | 33 (9.76%) | 5 (4.67%) | 11 (12.22%) | 0 (0.00%) | 2 (9.09%) | 3 (13.04%) | 0 (0.00%) | 0 (0.00%) | 5 (6.17%) | 12 (20.00%) |
| Guidelines | 69 (20.41%) | 27 (25.23%) | 24 (26.67%) | 7 (31.82%) | 8 (36.36%) | 8 (34.78%) | 0 (0.00%) | 4 (20.00%) | 5 (6.17%) | 13 (21.67%) |
| Other | 4 (1.18%) | 0 (0.00%) | 3 (3.33%) | 0 (0.00%) | 0 (0.00%) | 0 (0.00%) | 0 (0.00%) | 0 (0.00%) | 1 (1.23%) | 0 (0.00%) |
| Key: ECOG – Eastern Cooperative Oncology Group, EU5 – overall results from across France/Germany/Italy/Spain/UK, Physicians – healthcare providers, HRRm – homologous recombination repair genes, nmCRPC – non-metastatic castration-resistant prostate cancer, NHA- next-generation hormonal agents, PARPi- poly-adenosine diphosphate-ribose polymerase inhibitors, PS – performance status, PSA – prostate-specific antigen, PSADT – PSA doubling time. | | | | | | | | | | |

**Supplementary Table 8. Most common characteristics associated with the decision to treat patients with mHSPC with ADT + NHAs ADT + taxane-based chemotherapy and ADT alone according to physicians (overall and by country)**

|  | **Overall** | **EU5** | **USA** | **France** | **Germany** | **Italy** | **Spain** | **UK** | **Japan** | **China** |
| --- | --- | --- | --- | --- | --- | --- | --- | --- | --- | --- |
| Oncologists and urologists: | **N (%=338)** | **N (%=107)** | **N (%=90)** | **N (%=22)** | **N (%=22)** | **N (%=23)** | **N (%=20)** | **N (%=20)** | **N (%=81)** | **N (%=60)** |
| **Characteristics associated with the decision to treat mHSPC patients with ADT alone** | | | | | | | | | | |
| Age | 102 (30.18%) | 42 (39.25%) | 28 (31.11%) | 6 (27.27%) | 10 (45.45%) | 7 (30.43%) | 11 (55.00%) | 8 (40.00%) | 20 (24.69%) | 12 (20.00%) |
| ≤65 years | 18 (5.33%) | 6 (5.61%) | 5 (5.56%) | 1 (4.55%) | 1 (4.55%) | 1 (4.35%) | 2 (10.00%) | 1 (5.00%) | 4 (4.94%) | 3 (5.00%) |
| 66-79 years | 15 (4.44%) | 4 (3.74%) | 2 (2.22%) | 0 (0.00%) | 1 (4.55%) | 2 (8.70%) | 0 (0.00%) | 1 (5.00%) | 5 (6.17%) | 4 (6.67%) |
| ≥80 years | 69 (20.41%) | 32 (29.91%) | 21 (23.33%) | 5 (22.73%) | 8 (36.36%) | 4 (17.39%) | 9 (45.00%) | 6 (30.00%) | 11 (13.58%) | 5 (8.33%) |
| Fewer and less severe comorbidities | 120 (35.50%) | 27 (25.23%) | 33 (36.67%) | 2 (9.09%) | 6 (27.27%) | 8 (34.78%) | 4 (20.00%) | 7 (35.00%) | 35 (43.21%) | 25 (41.67%) |
| Toxicity profile of treatment (can tolerate adverse events) | 104 (30.77%) | 28 (26.17%) | 27 (30.00%) | 3 (13.64%) | 6 (27.27%) | 3 (13.04%) | 5 (25.00%) | 11 (55.00%) | 22 (27.16%) | 27 (45.00%) |
| Efficacy of treatment | 135 (39.94%) | 40 (37.38%) | 36 (40.00%) | 6 (27.27%) | 4 (18.18%) | 13 (56.52%) | 9 (45.00%) | 8 (40.00%) | 25 (30.86%) | 34 (56.67%) |
| Lower cost of treatment | 78 (23.08%) | 21 (19.63%) | 21 (23.33%) | 3 (13.64%) | 2 (9.09%) | 2 (8.70%) | 7 (35.00%) | 7 (35.00%) | 16 (19.75%) | 20 (33.33%) |
| Ease of administration | 110 (32.54%) | 38 (35.51%) | 28 (31.11%) | 8 (36.36%) | 6 (27.27%) | 9 (39.13%) | 6 (30.00%) | 9 (45.00%) | 17 (20.99%) | 27 (45.00%) |
| Covered under reimbursement policy | 62 (18.34%) | 14 (13.08%) | 23 (25.56%) | 1 (4.55%) | 5 (22.73%) | 4 (17.39%) | 2 (10.00%) | 2 (10.00%) | 5 (6.17%) | 20 (33.33%) |
| ECOG performance status | 51 (15.09%) | 18 (16.82%) | 24 (26.67%) | 3 (13.64%) | 3 (13.64%) | 0 (0.00%) | 7 (35.00%) | 5 (25.00%) | 3 (3.70%) | 6 (10.00%) |
| ECOG PS 0-1 | 14 (4.14%) | 1 (0.93%) | 9 (10.00%) | 0 (0.00%) | 0 (0.00%) | 0 (0.00%) | 0 (0.00%) | 1 (5.00%) | 1 (1.23%) | 3 (5.00%) |
| ECOG PS 2-4 | 37 (10.95%) | 17 (15.89%) | 15 (16.67%) | 3 (13.64%) | 3 (13.64%) | 0 (0.00%) | 7 (35.00%) | 4 (20.00%) | 2 (2.47%) | 3 (5.00%) |
| Patient choice | 71 (21.01%) | 16 (14.95%) | 23 (25.56%) | 3 (13.64%) | 5 (22.73%) | 4 (17.39%) | 2 (10.00%) | 2 (10.00%) | 11 (13.58%) | 21 (35.00%) |
| High Gleason score | 41 (12.13%) | 17 (15.89%) | 9 (10.00%) | 1 (4.55%) | 8 (36.36%) | 3 (13.04%) | 3 (15.00%) | 2 (10.00%) | 9 (11.11%) | 6 (10.00%) |
| High baseline PSA level | 39 (11.54%) | 12 (11.21%) | 4 (4.44%) | 1 (4.55%) | 3 (13.64%) | 5 (21.74%) | 0 (0.00%) | 3 (15.00%) | 17 (20.99%) | 6 (10.00%) |
| Shorter PSADT | 20 (5.92%) | 8 (7.48%) | 3 (3.33%) | 1 (4.55%) | 5 (22.73%) | 2 (8.70%) | 0 (0.00%) | 0 (0.00%) | 6 (7.41%) | 3 (5.00%) |
| ≤3 months | 10 (2.96%) | 4 (3.74%) | 2 (2.22%) | 0 (0.00%) | 2 (9.09%) | 2 (8.70%) | 0 (0.00%) | 0 (0.00%) | 2 (2.47%) | 2 (3.33%) |
| ≤6 months | 10 (2.96%) | 4 (3.74%) | 1 (1.11%) | 1 (4.55%) | 3 (13.64%) | 0 (0.00%) | 0 (0.00%) | 0 (0.00%) | 4 (4.94%) | 1 (1.67%) |
| >6 months | 0 (0.00%) | 0 (0.00%) | 0 (0.00%) | 0 (0.00%) | 0 (0.00%) | 0 (0.00%) | 0 (0.00%) | 0 (0.00%) | 0 (0.00%) | 0 (0.00%) |
| Prior treatment | 46 (13.61%) | 14 (13.08%) | 8 (8.89%) | 1 (4.55%) | 4 (18.18%) | 4 (17.39%) | 2 (10.00%) | 3 (15.00%) | 11 (13.58%) | 13 (21.67%) |
| High volume disease | 27 (7.99%) | 7 (6.54%) | 6 (6.67%) | 0 (0.00%) | 2 (9.09%) | 2 (8.70%) | 0 (0.00%) | 3 (15.00%) | 5 (6.17%) | 9 (15.00%) |
| Metastases of organ | 19 (5.62%) | 6 (5.61%) | 5 (5.56%) | 0 (0.00%) | 1 (4.55%) | 3 (13.04%) | 0 (0.00%) | 2 (10.00%) | 4 (4.94%) | 4 (6.67%) |
| Guidelines | 56 (16.57%) | 26 (24.30%) | 14 (15.56%) | 6 (27.27%) | 6 (27.27%) | 7 (30.43%) | 1 (5.00%) | 6 (30.00%) | 5 (6.17%) | 11 (18.33%) |
| Other | 9 (2.66%) | 3 (2.80%) | 5 (5.56%) | 2 (9.09%) | 1 (4.55%) | 0 (0.00%) | 0 (0.00%) | 0 (0.00%) | 1 (1.23%) | 0 (0.00%) |
| **Characteristics associated with the decision to treat mHSPC patients with ADT + NHA** | | | | | | | | | | |
| Age | 71 (21.01%) | 26 (24.30%) | 17 (18.89%) | 2 (9.09%) | 6 (27.27%) | 5 (21.74%) | 5 (25.00%) | 8 (40.00%) | 14 (17.28%) | 14 (23.33%) |
| ≤65 years | 32 (9.47%) | 9 (8.41%) | 7 (7.78%) | 0 (0.00%) | 1 (4.55%) | 2 (8.70%) | 3 (15.00%) | 3 (15.00%) | 8 (9.88%) | 8 (13.33%) |
| 66-79 years | 35 (10.36%) | 16 (14.95%) | 8 (8.89%) | 2 (9.09%) | 4 (18.18%) | 3 (13.04%) | 2 (10.00%) | 5 (25.00%) | 5 (6.17%) | 6 (10.00%) |
| ≥80 years | 4 (1.18%) | 1 (0.93%) | 2 (2.22%) | 0 (0.00%) | 1 (4.55%) | 0 (0.00%) | 0 (0.00%) | 0 (0.00%) | 1 (1.23%) | 0 (0.00%) |
| Fewer and less severe comorbidities | 100 (29.59%) | 29 (27.10%) | 17 (18.89%) | 4 (18.18%) | 8 (36.36%) | 8 (34.78%) | 2 (10.00%) | 7 (35.00%) | 25 (30.86%) | 29 (48.33%) |
| Toxicity profile of treatment (can tolerate adverse events) | 95 (28.11%) | 29 (27.10%) | 19 (21.11%) | 4 (18.18%) | 8 (36.36%) | 5 (21.74%) | 7 (35.00%) | 5 (25.00%) | 23 (28.40%) | 24 (40.00%) |
| Efficacy of treatment | 192 (56.80%) | 61 (57.01%) | 63 (70.00%) | 13 (59.09%) | 10 (45.45%) | 13 (56.52%) | 17 (85.00%) | 8 (40.00%) | 32 (39.51%) | 36 (60.00%) |
| Lower cost of treatment | 45 (13.31%) | 7 (6.54%) | 14 (15.56%) | 0 (0.00%) | 1 (4.55%) | 5 (21.74%) | 1 (5.00%) | 0 (0.00%) | 12 (14.81%) | 12 (20.00%) |
| Ease of administration | 78 (23.08%) | 26 (24.30%) | 19 (21.11%) | 5 (22.73%) | 7 (31.82%) | 5 (21.74%) | 5 (25.00%) | 4 (20.00%) | 12 (14.81%) | 21 (35.00%) |
| Covered under reimbursement policy | 59 (17.46%) | 20 (18.69%) | 14 (15.56%) | 3 (13.64%) | 5 (22.73%) | 4 (17.39%) | 7 (35.00%) | 1 (5.00%) | 10 (12.35%) | 15 (25.00%) |
| ECOG performance status | 43 (12.72%) | 17 (15.89%) | 18 (20.00%) | 1 (4.55%) | 4 (18.18%) | 1 (4.35%) | 4 (20.00%) | 7 (35.00%) | 3 (3.70%) | 5 (8.33%) |
| ECOG PS 0-1 | 36 (10.65%) | 13 (12.15%) | 16 (17.78%) | 1 (4.55%) | 3 (13.64%) | 0 (0.00%) | 4 (20.00%) | 5 (25.00%) | 3 (3.70%) | 4 (6.67%) |
| ECOG PS2-4 | 7 (2.07%) | 4 (3.74%) | 2 (2.22%) | 0 (0.00%) | 1 (4.55%) | 1 (4.35%) | 0 (0.00%) | 2 (10.00%) | 0 (0.00%) | 1 (1.67%) |
| Patient choice | 61 (18.05%) | 18 (16.82%) | 17 (18.89%) | 2 (9.09%) | 4 (18.18%) | 3 (13.04%) | 3 (15.00%) | 6 (30.00%) | 11 (13.58%) | 15 (25.00%) |
| High Gleason score | 86 (25.44%) | 30 (28.04%) | 22 (24.44%) | 5 (22.73%) | 9 (40.91%) | 6 (26.09%) | 5 (25.00%) | 5 (25.00%) | 19 (23.46%) | 15 (25.00%) |
| High baseline PSA level | 67 (19.82%) | 20 (18.69%) | 13 (14.44%) | 2 (9.09%) | 3 (13.64%) | 4 (17.39%) | 6 (30.00%) | 5 (25.00%) | 16 (19.75%) | 18 (30.00%) |
| Shorter PSADTNHA | 35 (10.36%) | 10 (9.35%) | 12 (13.33%) | 2 (9.09%) | 2 (9.09%) | 2 (8.70%) | 2 (10.00%) | 2 (10.00%) | 5 (6.17%) | 8 (13.33%) |
| ≤3 months | 14 (4.14%) | 3 (2.80%) | 3 (3.33%) | 0 (0.00%) | 1 (4.55%) | 1 (4.35%) | 0 (0.00%) | 1 (5.00%) | 1 (1.23%) | 7 (11.67%) |
| ≤6 months | 21 (6.21%) | 7 (6.54%) | 9 (10.00%) | 2 (9.09%) | 1 (4.55%) | 1 (4.35%) | 2 (10.00%) | 1 (5.00%) | 4 (4.94%) | 1 (1.67%) |
| >6 months | 0 (0.00%) | 0 (0.00%) | 0 (0.00%) | 0 (0.00%) | 0 (0.00%) | 0 (0.00%) | 0 (0.00%) | 0 (0.00%) | 0 (0.00%) | 0 (0.00%) |
| Prior treatment | 49 (14.50%) | 17 (15.89%) | 11 (12.22%) | 2 (9.09%) | 4 (18.18%) | 4 (17.39%) | 2 (10.00%) | 5 (25.00%) | 7 (8.64%) | 14 (23.33%) |
| High volume disease | 59 (17.46%) | 18 (16.82%) | 12 (13.33%) | 3 (13.64%) | 3 (13.64%) | 4 (17.39%) | 2 (10.00%) | 6 (30.00%) | 11 (13.58%) | 18 (30.00%) |
| Metastases of organ | 56 (16.57%) | 17 (15.89%) | 9 (10.00%) | 3 (13.64%) | 4 (18.18%) | 3 (13.04%) | 3 (15.00%) | 4 (20.00%) | 17 (20.99%) | 13 (21.67%) |
| Guidelines | 74 (21.89%) | 30 (28.04%) | 27 (30.00%) | 11 (50.00%) | 6 (27.27%) | 7 (30.43%) | 2 (10.00%) | 4 (20.00%) | 6 (7.41%) | 11 (18.33%) |
| Other | 4 (1.18%) | 0 (0.00%) | 3 (3.33%) | 0 (0.00%) | 0 (0.00%) | 0 (0.00%) | 0 (0.00%) | 0 (0.00%) | 1 (1.23%) | 0 (0.00%) |
| **Characteristics associated with the decision to treat mHSPC patients with ADT + taxane-based chemotherapy** | | | | | | | | | | |
| Age | 72 (21.30%) | 29 (27.10%) | 23 (16.31%) | 20 (22.22%) | 5 (22.73%) | 6 (27.27%) | 4 (17.39%) | 6 (30.00%) | 8 (40.00%) | 14 (17.28%) |
| ≤65 years | 59 (17.46%) | 23 (21.50%) | 21 (14.89%) | 15 (16.67%) | 4 (18.18%) | 4 (18.18%) | 2 (8.70%) | 6 (30.00%) | 7 (35.00%) | 12 (14.81%) |
| 66-79 years | 12 (3.55%) | 5 (4.67%) | 2 (1.42%) | 5 (5.56%) | 1 (4.55%) | 1 (4.55%) | 2 (8.70%) | 0 (0.00%) | 1 (5.00%) | 2 (2.47%) |
| ≥80 years | 1 (0.30%) | 1 (0.93%) | 0 (0.00%) | 0 (0.00%) | 0 (0.00%) | 1 (4.55%) | 0 (0.00%) | 0 (0.00%) | 0 (0.00%) | 0 (0.00%) |
| Fewer and less severe comorbidities | 81 (23.96%) | 28 (26.17%) | 36 (25.53%) | 17 (18.89%) | 5 (22.73%) | 8 (36.36%) | 5 (21.74%) | 2 (10.00%) | 8 (40.00%) | 21 (25.93%) |
| Toxicity profile of treatment (can tolerate adverse events) | 90 (26.63%) | 32 (29.91%) | 38 (26.95%) | 20 (22.22%) | 1 (4.55%) | 6 (27.27%) | 5 (21.74%) | 7 (35.00%) | 13 (65.00%) | 19 (23.46%) |
| Efficacy of treatment | 149 (44.08%) | 39 (36.45%) | 66 (46.81%) | 44 (48.89%) | 5 (22.73%) | 9 (40.91%) | 7 (30.43%) | 8 (40.00%) | 10 (50.00%) | 33 (40.74%) |
| Lower cost of treatment | 63 (18.64%) | 17 (15.89%) | 33 (23.40%) | 13 (14.44%) | 2 (9.09%) | 3 (13.64%) | 6 (26.09%) | 3 (15.00%) | 3 (15.00%) | 5 (6.17%) |
| Ease of administration | 45 (13.31%) | 11 (10.28%) | 26 (18.44%) | 8 (8.89%) | 1 (4.55%) | 2 (9.09%) | 4 (17.39%) | 3 (15.00%) | 1 (5.00%) | 9 (11.11%) |
| Covered under reimbursement policy | 48 (14.20%) | 11 (10.28%) | 26 (18.44%) | 11 (12.22%) | 1 (4.55%) | 5 (22.73%) | 2 (8.70%) | 3 (15.00%) | 0 (0.00%) | 6 (7.41%) |
| ECOG performance status | 50 (14.79%) | 16 (14.95%) | 15 (10.64%) | 19 (21.11%) | 3 (13.64%) | 3 (13.64%) | 0 (0.00%) | 6 (30.00%) | 4 (20.00%) | 5 (6.17%) |
| ECOG PS 0-1 | 43 (12.72%) | 15 (14.02%) | 10 (7.09%) | 18 (20.00%) | 3 (13.64%) | 2 (9.09%) | 0 (0.00%) | 6 (30.00%) | 4 (20.00%) | 1 (1.23%) |
| ECOG PS 2-4 | 7 (2.07%) | 1 (0.93%) | 5 (3.55%) | 1 (1.11%) | 0 (0.00%) | 1 (4.55%) | 0 (0.00%) | 0 (0.00%) | 0 (0.00%) | 4 (4.94%) |
| Patient choice | 49 (14.50%) | 10 (9.35%) | 21 (14.89%) | 18 (20.00%) | 0 (0.00%) | 3 (13.64%) | 2 (8.70%) | 0 (0.00%) | 5 (25.00%) | 8 (9.88%) |
| High Gleason score | 94 (27.81%) | 35 (32.71%) | 31 (21.99%) | 28 (31.11%) | 7 (31.82%) | 11 (50.00%) | 5 (21.74%) | 7 (35.00%) | 5 (25.00%) | 18 (22.22%) |
| High baseline PSA level | 66 (19.53%) | 24 (22.43%) | 26 (18.44%) | 16 (17.78%) | 2 (9.09%) | 8 (36.36%) | 3 (13.04%) | 7 (35.00%) | 4 (20.00%) | 11 (13.58%) |
| Shorter PSADT | 44 (13.02%) | 16 (14.95%) | 12 (8.51%) | 16 (17.78%) | 3 (13.64%) | 5 (22.73%) | 4 (17.39%) | 2 (10.00%) | 2 (10.00%) | 6 (7.41%) |
| ≤3 months | 23 (6.80%) | 9 (8.41%) | 7 (4.96%) | 7 (7.78%) | 2 (9.09%) | 1 (4.55%) | 4 (17.39%) | 0 (0.00%) | 2 (10.00%) | 3 (3.70%) |
| ≤6 months | 21 (6.21%) | 7 (6.54%) | 5 (3.55%) | 9 (10.00%) | 1 (4.55%) | 4 (18.18%) | 0 (0.00%) | 2 (10.00%) | 0 (0.00%) | 3 (3.70%) |
| >6 months | 0 (0.00%) | 0 (0.00%) | 0 (0.00%) | 0 (0.00%) | 0 (0.00%) | 0 (0.00%) | 0 (0.00%) | 0 (0.00%) | 0 (0.00%) | 0 (0.00%) |
| Prior treatment | 60 (17.75%) | 20 (18.69%) | 26 (18.44%) | 14 (15.56%) | 2 (9.09%) | 5 (22.73%) | 5 (21.74%) | 3 (15.00%) | 5 (25.00%) | 11 (13.58%) |
| High volume disease | 111 (32.84%) | 44 (41.12%) | 33 (23.40%) | 34 (37.78%) | 9 (40.91%) | 9 (40.91%) | 10 (43.48%) | 10 (50.00%) | 6 (30.00%) | 16 (19.75%) |
| Metastases of organ | 103 (30.47%) | 42 (39.25%) | 37 (26.24%) | 24 (26.67%) | 9 (40.91%) | 11 (50.00%) | 7 (30.43%) | 10 (50.00%) | 5 (25.00%) | 20 (24.69%) |
| Guidelines | 62 (18.34%) | 20 (18.69%) | 23 (16.31%) | 19 (21.11%) | 3 (13.64%) | 8 (36.36%) | 6 (26.09%) | 0 (0.00%) | 3 (15.00%) | 8 (9.88%) |
| Other | 9 (2.66%) | 1 (0.93%) | 2 (1.42%) | 6 (6.67%) | 0 (0.00%) | 0 (0.00%) | 0 (0.00%) | 1 (5.00%) | 0 (0.00%) | 2 (2.47%) |
| Key: ECOG – Eastern Cooperative Oncology Group, EU5 – overall results from across France/Germany/Italy/Spain/UK, Physicians – healthcare providers, HRRm – homologous recombination repair genes, mHSPC – metastatic hormone-sensitive prostate cancer, NHA- next-generation hormonal agents, PARPi- poly-adenosine diphosphate-ribose polymerase inhibitors, PS – performance status, PSA – prostate-specific antigen, PSADT – PSA doubling time. | | | | | | | | | | |


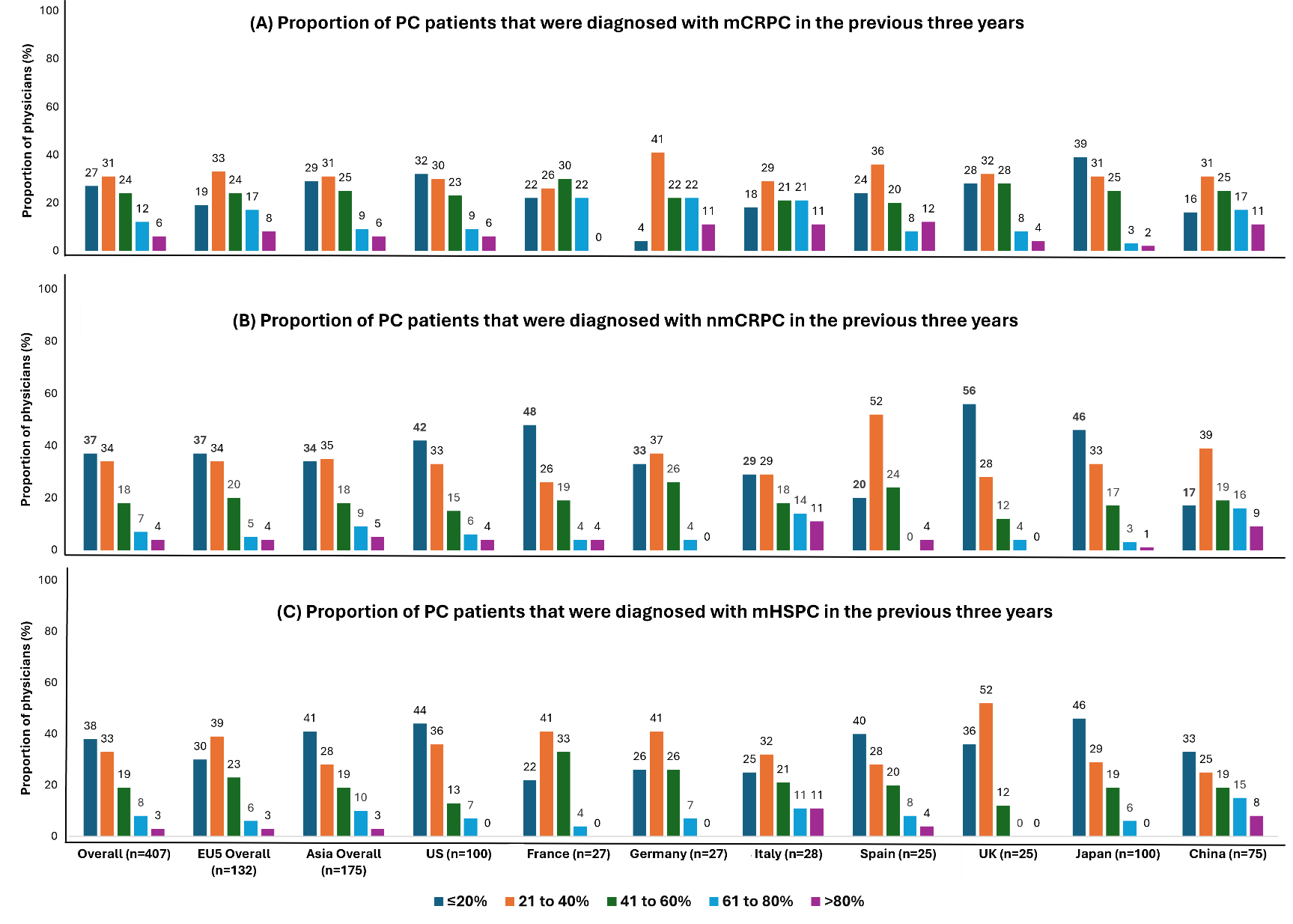


**Supplementary Figure 1.** Proportion of prostate cancer patients that were diagnosed with mCRPC, nmCRPC, or mHSPC in the previous three years overall and by country/region. Key: HRRm – homologous recombination repair genes, mCRPC – metastatic castration-resistant prostate cancer, mHSPC –metastatic hormone-sensitive prostate cancer, nmCRPC – non-metastatic castration-resistant prostate cancer, PC – prostate cancer, UK – United Kingdom.

**Supplementary Figure 2.** The time point at which HRRm testing is prescribed as reported by physicians.

Key: HRRm – homologous recombination repair genes, mCRPC – metastatic castration-resistant prostate cancer, mHSPC –metastatic hormone-sensitive prostate cancer, nmCRPC – non-metastatic castration-resistant prostate cancer, PC – prostate cancer, UK – United Kingdom.

**Supplementary Figure 3.** First and second tests considered for HRRm testing as reported by physicians.

Key: ctDNA – circulating tumor DNA, EU5 – overall results from across France/Germany/Italy/Spain/UK, HRRm – homologous recombination repair genes, UK – United Kingdom. USA – United States.

**
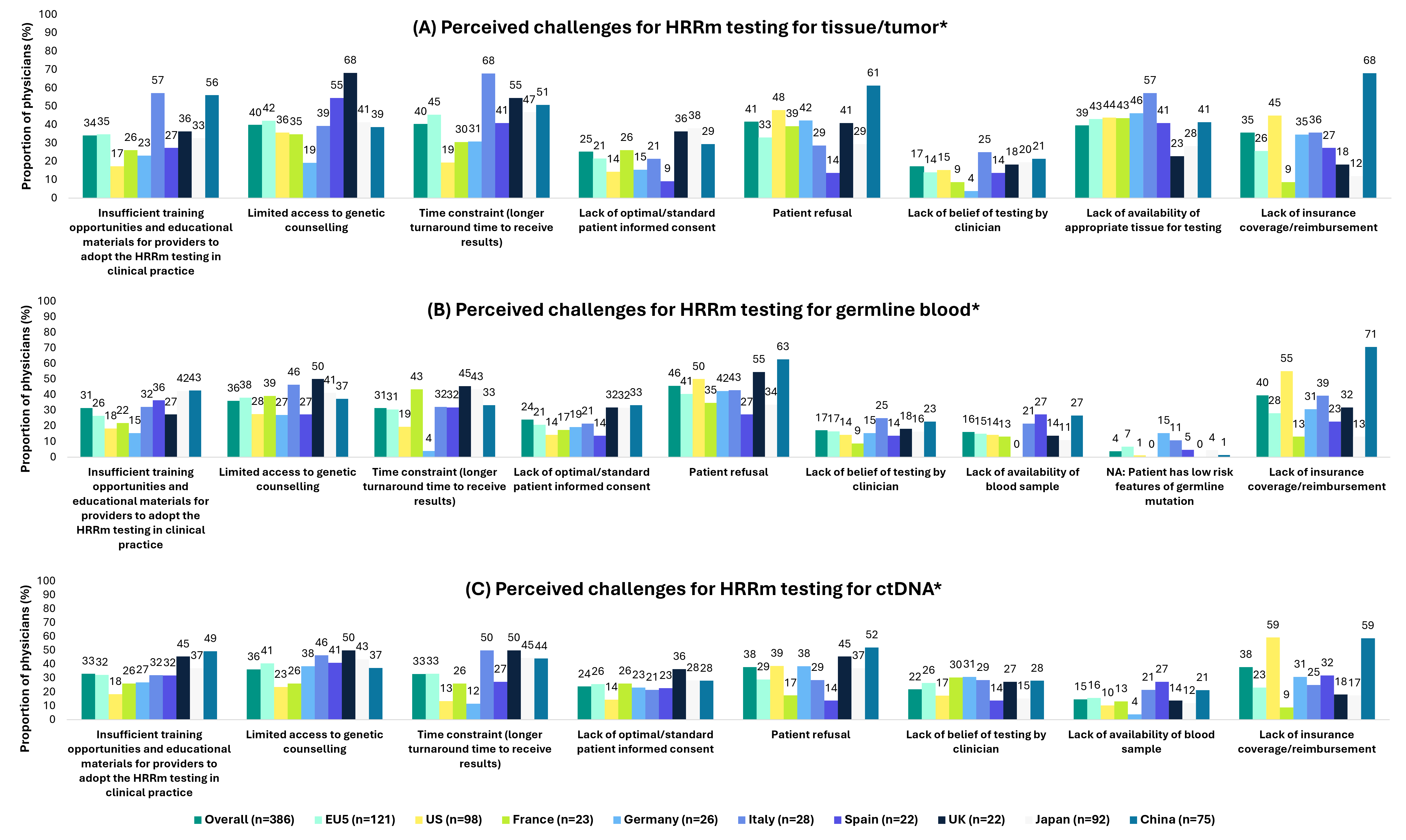
**

**Supplementary Figure 4.** Perceived challenges for HRRm. *Responses not mutually exclusive (respondents selected all options that applied). Key: ctDNA – circulating tumor DNA, EU5 – overall results from across France/Germany/Italy/Spain/UK, HRRm – homologous recombination repair genes, UK – United Kingdom. USA – United States
